# Oxidative Stress and Inflammatory Cytokines as Drivers of Cardiac Remodeling in COPD Patients

**DOI:** 10.3390/antiox15091141

**Published:** 2026-09-09

**Authors:** Elena-Andreea Moaleș, Lucia Corina Dima-Cozma, Cristina Andreea Adam, Doina-Clementina Cojocaru, Cristina Gena Dascălu, Andreea Iațentiuc, Iustin Mihai Iațentiuc, Tatiana Drâmbă, Maura Gabriela Felea, Robert Negru, Ioana Mădălina Zota, Romică Sebastian Cozma, Maria Magdalena Leon, Florin Mitu

**Affiliations:** 1 ”Grigore T. Popa” University of Medicine and Pharmacy, University Street No. 16, 700115 Iași, Romania; 2 Elytis Hospital Iasi, Gheorghe Săulescu Street No. 43 A, 700010 Iasi, Romania; 3 Clinical Rehabilitation Hospital, Pantelimon Halipa Street No. 14, 700661 Iași, Romania; 4 Romanian Academy of Scientists, 050045 Bucharest, Romania; 5 Romanian Academy of Medical Sciences, 030167 Bucharest, Romania

**Keywords:** oxidative stress, inflammatory cytokines, cardiac remodeling, COPD

## Abstract

Chronic obstructive pulmonary disease (COPD) is increasingly recognized as a systemic disease characterized by chronic inflammation and oxidative stress, with frequent cardiovascular involvement. This study investigated the associations between oxidative stress-related inflammatory markers and structural and electrical cardiac abnormalities in patients with COPD using a comprehensive multimodal cardiopulmonary assessment. A total of 100 clinically stable patients with COPD underwent inflammatory biomarker evaluation (IL-6, IL-8, IL-1β, and TNF-α), transthoracic echocardiography and 24 h Holter ECG monitoring. Advanced COPD was associated with significant differences in several structural cardiac parameters, including left atrial volume (*p* = 0.016), left atrial height (*p* = 0.027), and selected left ventricular parameters, while left ventricular ejection fraction remained preserved. Electrical abnormalities were significantly associated with systemic inflammatory markers. SII showed the highest discriminatory performance for sinus rhythm abnormalities (AUC = 0.736), whereas IL-6 showed the highest discriminatory performance for ventricular premature complexes (AUC = 0.732). Ventricular ectopic activity was also associated with significantly higher SII, SIRI, MLR, NLR, and PLR. Systemic inflammatory markers were associated with structural and electrical cardiac abnormalities in COPD, suggesting that they may provide complementary information regarding cardiovascular involvement. However, given the cross-sectional design and moderate discriminatory performance of these biomarkers, the findings should be considered exploratory and do not establish causal relationships. Prospective studies are needed to determine their potential role in cardiovascular risk stratification.

## 1. Introduction

Chronic obstructive pulmonary disease (COPD) has traditionally been defined by persistent airflow limitation and chronic respiratory symptoms; however, growing evidence suggests that its clinical impact extends far beyond the lungs [1,2]. COPD is currently recognized as a complex systemic disease in which chronic inflammation, oxidative stress, and vascular dysfunction contribute to the development of multiple extrapulmonary manifestations [3]. In recent years, cardiovascular involvement has become an important area of interest, given its major influence on quality of life, hospitalization rates, and overall mortality among COPD patients [4,5].

**Figure 1:**
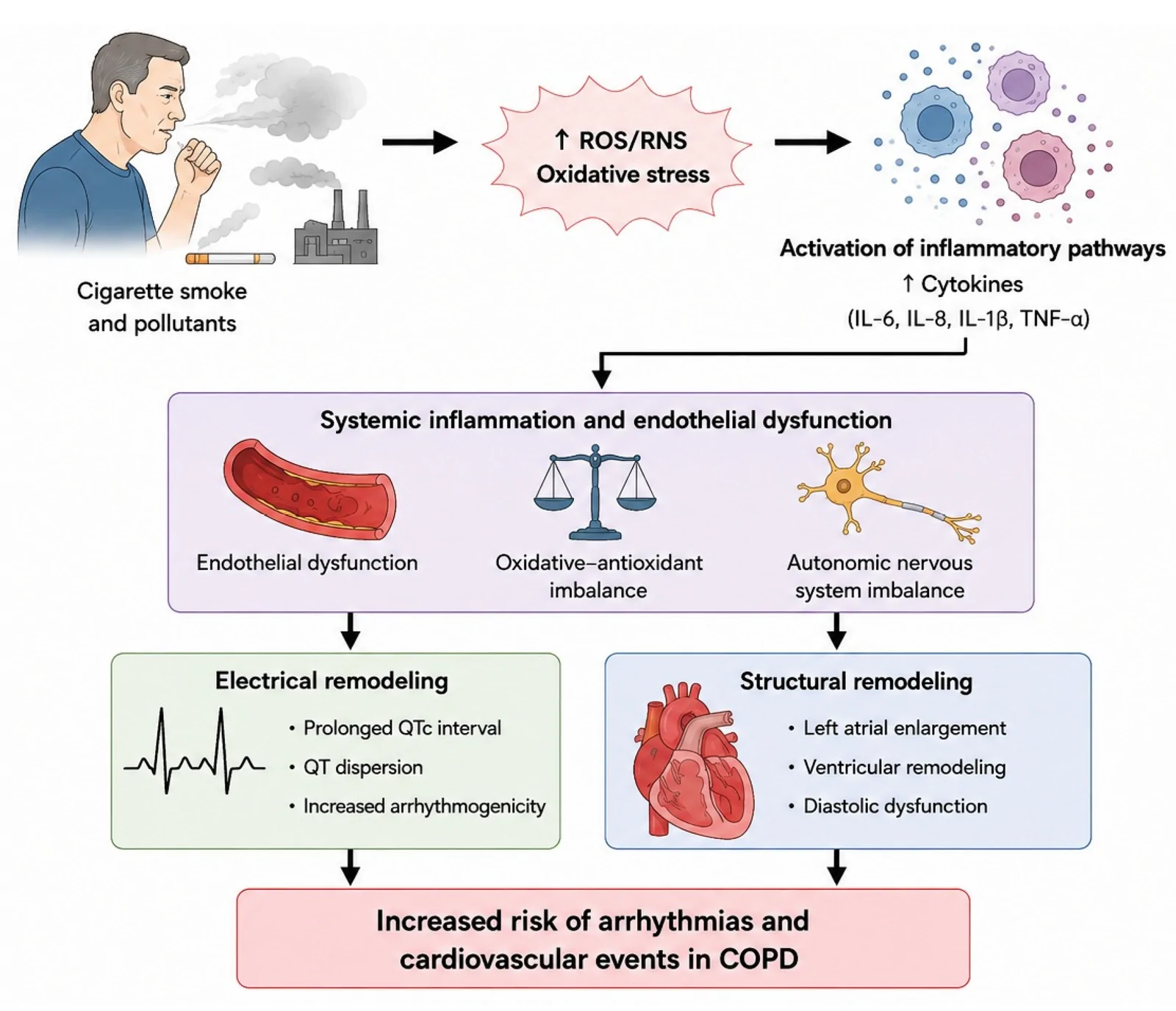
Proposed pathophysiological mechanisms linking oxidative stress and systemic inflammation to cardiac remodeling in COPD. Cigarette smoke and environmental pollutants promote oxidative stress and the activation of inflammatory pathways, leading to increased release of pro-inflammatory cytokines. The resulting systemic inflammation, endothelial dysfunction, oxidative–antioxidant imbalance, and autonomic nervous system dysregulation may contribute to both electrical and structural cardiac remodeling, ultimately increasing the risk of arrhythmias and cardiovascular events in patients with COPD. Abbreviations: COPD, chronic obstructive pulmonary disease; ROS, reactive oxygen species; RNS, reactive nitrogen species; IL, interleukin; TNF-α, tumor necrosis factor α; QTc, corrected QT interval.

Among the mechanisms implicated in COPD pathogenesis, oxidative stress appears to play a central role [6]. Figure 1 illustrates the proposed mechanisms linking oxidative stress and inflammatory cytokine activation to electrical and structural cardiac remodeling in COPD patients. Continuous exposure to cigarette smoke and other inhaled toxic particles promotes excessive generation of reactive oxygen and nitrogen species, leading to an imbalance between oxidant production and endogenous antioxidant defense systems [7,8]. This persistent redox imbalance contributes not only to airway inflammation and tissue destruction, but also to systemic endothelial injury and chronic low-grade inflammation [9]. As a consequence, several pro-inflammatory cytokines, including interleukin-6 (IL-6), interleukin-8 (IL-8) and tumor necrosis factor (TNF)-α, become increasingly expressed and may further amplify both pulmonary and cardiovascular damage [10,11]. Recent evidence suggests that IL-1 beta may represent a key mediator linking oxidative stress to persistent systemic inflammation and cardiovascular remodeling in COPD patients [12].

The relationship between oxidative stress and cardiovascular dysfunction in COPD is particularly complex. Chronic hypoxia, systemic inflammation, autonomic nervous system imbalance, and oxidative injury may collectively favor myocardial remodeling and vascular impairment [13,14,15]. Previous studies have shown that COPD patients frequently exhibit subclinical cardiac abnormalities, even in the absence of previously diagnosed cardiovascular disease [16]. Structural alterations involving both right and left ventricular function, pulmonary vascular changes, impaired heart rate variability, and different forms of supraventricular or ventricular arrhythmias have all been described in this population [17,18]. These findings suggest that cardiovascular involvement may develop early during disease evolution and remain insufficiently recognized in routine clinical practice.

Inflammatory cytokines are increasingly considered important mediators linking oxidative stress to cardiovascular impairment in COPD [19]. Elevated circulating cytokine levels have been associated with disease severity, exacerbation frequency, reduced exercise capacity, and adverse cardiovascular outcomes [20]. At the same time, advances in multimodal cardiac assessment, including electrocardiographic Holter monitoring and echocardiography, have created new opportunities for identifying early electrical and structural cardiac changes in COPD patients [21]. Nevertheless, the interaction between systemic inflammatory burden, oxidative stress, and cardiac remodeling remains incompletely understood.

In this context, the present study aimed to evaluate the relationship between oxidative stress-related inflammatory cytokines and cardiac remodeling in patients with COPD through a multimodal cardiopulmonary assessment. By correlating inflammatory biomarkers with electrocardiographic and echocardiographic findings, we sought to better characterize the mechanisms involved in cardiovascular impairment associated with COPD and to identify potential indicators of early subclinical cardiac dysfunction [22,23].

## 2. Materials and Methods

### 2.1. Study Design and Population

This study is an observational cross-sectional investigation conducted in a cohort of patients diagnosed with chronic obstructive pulmonary disease (COPD) who were evaluated at the Clinical Rehabilitation Hospital Iasi. The study was designed to investigate the relationship between oxidative stress-associated inflammatory cytokines and cardiac remodeling through a comprehensive multimodal cardiopulmonary assessment integrating inflammatory biomarkers, pulmonary function testing, ambulatory electrocardiographic monitoring, and transthoracic echocardiography.

Patients were consecutively recruited during routine outpatient and inpatient clinical assessments. The diagnosis of COPD was established in accordance with the Global Initiative for Chronic Obstructive Lung Disease (GOLD) criteria, integrating clinical history, exposure to relevant risk factors, and the presence of persistent respiratory symptoms, and was subsequently confirmed by post-bronchodilator spirometry demonstrating a forced vital capacity ratio (FEV1/FVC) below 0.70.

Patients were considered eligible for inclusion if they had a confirmed diagnosis of COPD according to GOLD criteria, were older than 35 years, and provided written informed consent prior to enrollment. Only clinically stable individuals without evidence of acute exacerbation or respiratory infection at the time of evaluation were included.

Exclusion criteria included active malignancy, major neurological or neuropsychiatric disorders, advanced renal or hepatic dysfunction, acute inflammatory or infectious diseases, and any condition potentially associated with significant alterations in systemic inflammatory status or circulating interleukin levels. Patients who declined participation or failed to provide written informed consent were also excluded from the study.

Beyond the primary objective of evaluating the relationship between oxidative stress-related inflammatory markers and structural and electrical cardiac remodeling in COPD, a secondary exploratory analysis was performed to assess sex-related differences in echocardiographic parameters and to identify potential sex-specific patterns of cardiac remodeling.

### 2.2. Clinical and Functional Assessment

All participants underwent a comprehensive clinical and functional respiratory evaluation performed during hospitalization. Dyspnea severity was systematically quantified using the modified Medical Research Council (mMRC) dyspnea scale, while the overall burden of respiratory symptoms and their impact on daily activities and quality of life were assessed using the COPD Assessment Test (CAT) questionnaire.

Peripheral oxygen saturation (SpO_2_) was measured under resting conditions by pulse oximetry after an adequate stabilization period. In addition, nocturnal oxygenation status was evaluated through overnight pulse oximetry monitoring, allowing the identification of nocturnal desaturation episodes and sleep-related oxygenation abnormalities that may not be evident during daytime assessment.

The functional respiratory evaluation also included spirometric parameters, clinical symptom profiling, and the assessment of hypoxia severity, aiming to provide an integrated characterization of disease burden and functional impairment in patients with COPD.

### 2.3. Inflammatory Profile and Oxidative Stress Evaluation

Given the central role of chronic systemic inflammation and oxidative stress in COPD pathogenesis and progression, serum levels of several pro-inflammatory cytokines were assessed, including interleukin (IL)-6, IL-8, IL-1β, and tumor necrosis factor-alpha (TNF-α). These biomarkers were selected because of their well-established involvement in persistent airway inflammation, oxidative imbalance, endothelial dysfunction, and cardiovascular remodeling associated with COPD.

Serum concentrations of IL-6, IL-8, IL-1β, and TNF-α were measured using commercially available human sandwich enzyme-linked immunosorbent assay (ELISA) kits obtained from antibodies-online (Aachen, Germany) according to the supplier’s instructions. The following kits were used: Human IL-6 ELISA Kit (Catalog No. ABIN365163; detection range, 7.8–500 pg/mL), Human IL-8 ELISA Kit (Catalog No. ABIN411305; detection range, 15.6–1000 pg/mL), Human IL-1β ELISA Kit (Catalog No. ABIN415066; detection range, 15.6–1000 pg/mL), and Human TNF-α ELISA Kit (Catalog No. ABIN411361; detection range, 15.6–1000 pg/mL). All assays were quantitative colorimetric sandwich ELISAs performed using human serum. All analyses were conducted at the Advanced Research and Development Center for Experimental Medicine “Prof. Ostin C. Mungiu” (CEMEX), Iași, Romania.

Cytokine concentrations were analyzed as continuous variables; therefore, no predefined clinical reference cutoffs were applied. Cutoff values reported in the Results were derived exclusively from receiver operating characteristic (ROC) curve analyses using the Youden index and were used to assess the discriminatory performance of selected biomarkers for specific electrocardiographic and arrhythmic abnormalities.

### 2.4. Cardiovascular Function Assessment

Considering the recognized interplay between chronic systemic inflammation, oxidative stress, and cardiovascular dysfunction in COPD, a comprehensive cardiovascular assessment was performed in all enrolled patients in order to identify structural and electrical cardiac abnormalities potentially associated with disease severity and systemic inflammatory burden.

Cardiovascular assessment included transthoracic echocardiography and 24 h ambulatory electrocardiographic monitoring (Holter ECG). Echocardiographic examination was conducted using standardized imaging protocols and provided information regarding left ventricular systolic and diastolic function, chamber dimensions, right ventricular performance, pulmonary artery pressure estimation, and the presence of structural cardiac remodeling. Parameters such as left ventricular ejection fraction (LVEF), left atrial diameter, right ventricular dimensions, and systolic pulmonary arterial pressure (sPAP) were analyzed in relation to inflammatory and oxidative stress markers.

The integration of inflammatory biomarkers with cardiovascular functional parameters aimed to provide a multidimensional characterization of the cardiopulmonary continuum in COPD, emphasizing the potential contribution of oxidative stress-related pathways to both structural and electrical cardiac impairment. All cardiovascular investigations were interpreted according to current international guideline recommendations and standardized clinical practice protocols.

**Figure 2:**
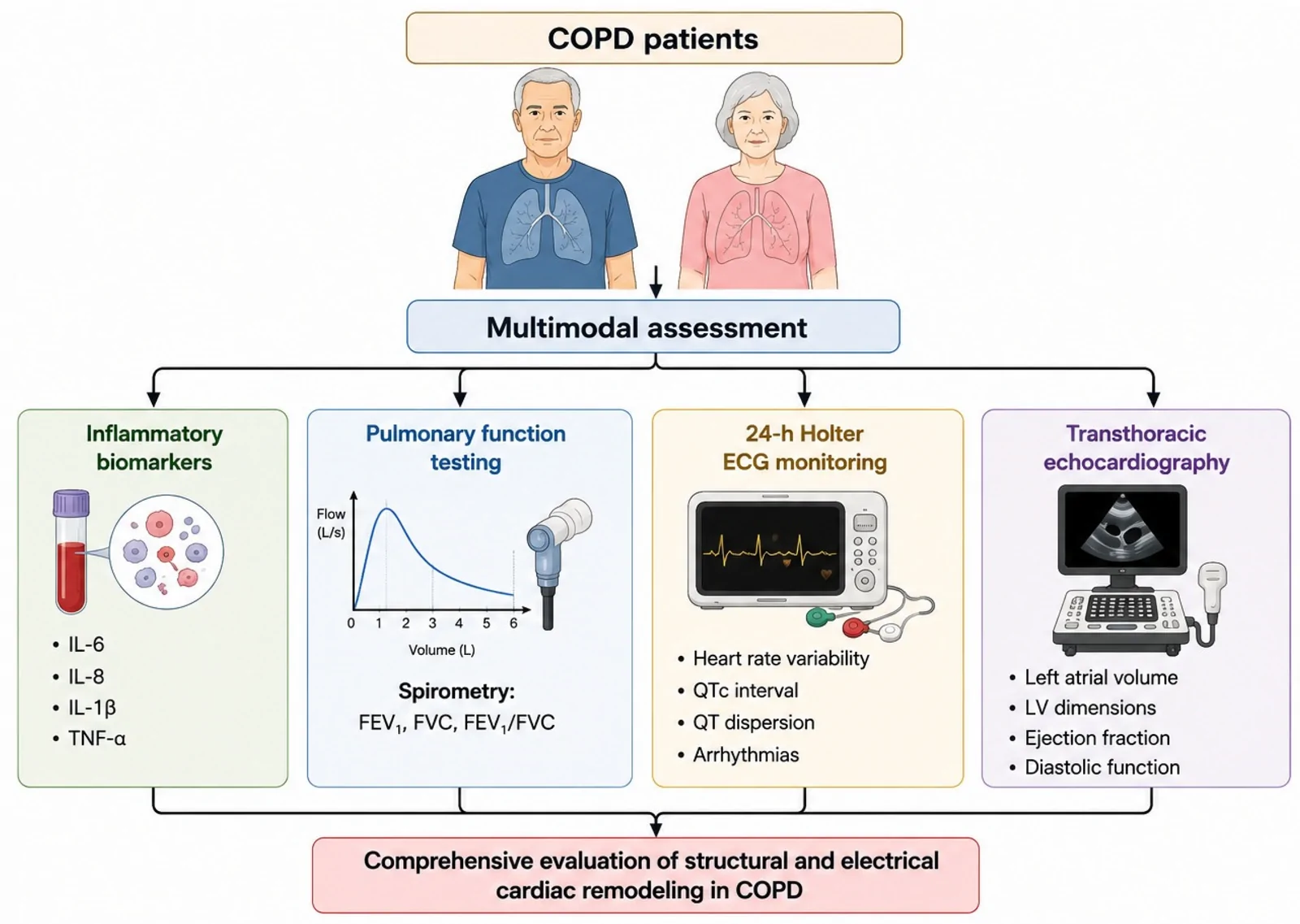
Multimodal cardiopulmonary assessment performed in the study. The integrated evaluation included inflammatory biomarker assessment, pulmonary function testing, 24 h Holter ECG monitoring, and transthoracic echocardiography, providing a comprehensive characterization of cardiovascular involvement in patients with COPD.

An overview of the multimodal cardiopulmonary assessment performed in the study, including inflammatory biomarker evaluation, pulmonary function testing, 24 h Holter ECG monitoring, and transthoracic echocardiography, is provided in Figure 2.

### 2.5. Statistical Analysis

Statistical analyses were performed using SPSS software, version 26.0 (IBM Corp., Chicago, IL, USA). Prior to group comparisons, the normality of continuous variables was assessed by means of the Shapiro–Wilk test. Normally distributed variables were expressed as mean ± standard deviation (SD) and compared using independent samples *t*-tests or one-way ANOVA, as appropriate, with Tukey HSD post hoc tests applied for pairwise comparisons following significant ANOVA results. For variables that did not meet the assumption of normality, results were additionally expressed as medians and interquartile ranges, and the Mann–Whitney or Kruskal–Wallis test was employed, with Bonferroni-adjusted pairwise post hoc comparisons performed when the Kruskal–Wallis test indicated significant differences among groups. Differences in categorical variables were evaluated using Pearson’s chi-squared test with Fisher’s correction.

Associations between inflammatory biomarkers and electrocardiographic parameters were assessed using Pearson’s correlation coefficient (r), with correlations classified as weak, moderate, or strong according to standard conventions.

Receiver operating characteristic (ROC) curve analyses were performed for inflammatory biomarkers and hematological indices showing statistically significant associations with specific electrocardiographic or arrhythmic abnormalities, in order to assess their discriminatory performance. The area under the curve (AUC), together with its 95% confidence interval (CI), was calculated, and optimal cutoff values were determined using the Youden index, with the corresponding sensitivity and specificity reported. Given the exploratory nature of the study, ROC-derived cutoff values were considered exploratory and were not interpreted as clinically validated diagnostic thresholds.

Post hoc pairwise comparisons following significant omnibus tests were adjusted for multiple comparisons using Tukey HSD after one-way ANOVA and Bonferroni correction after Kruskal–Wallis testing, as appropriate. No global adjustment for multiple testing was applied across the broader set of analyses involving inflammatory biomarkers, arrhythmic phenotypes, ROC curves, and correlation analyses. These analyses were considered exploratory; therefore, statistically significant findings should be interpreted cautiously and regarded as hypothesis-generating rather than confirmatory. Statistical significance was defined as *p* < 0.05, while *p* < 0.01 was considered indicative of a stronger level of statistical evidence.

Sample size adequacy was verified using G*Power 3.1.9.7 software based on the principal group-comparison analyses performed in the study. For chi-squared analyses of categorical variables with up to 6 degrees of freedom, assuming a large effect size (w = 0.5), an alpha level of 0.05, and a statistical power of 0.80, the minimum required sample size was estimated at 55 subjects. For comparisons of continuous cardiopulmonary and inflammatory parameters across the three COPD severity groups using one-way ANOVA, assuming a large effect size (f = 0.4), an alpha level of 0.05, and a power of 0.80, the minimum required sample size was 66 subjects. Accordingly, the study sample of 100 patients exceeded both thresholds and was considered adequate for the planned group-comparison analyses.

Sex-based comparisons of echocardiographic parameters were performed as a secondary exploratory analysis using the independent-samples *t*-test or Mann–Whitney U test, according to data distribution.

Exploratory multivariable logistic regression analyses were performed to assess whether the associations of SII and NLR with sinus rhythm abnormalities persisted after adjustment for clinically relevant potential confounders. Because SII and NLR exhibited right-skewed distributions, both biomarkers were log_2_-transformed before model fitting, allowing the adjusted odds ratios to be interpreted per doubling of the respective biomarker. The primary models were adjusted for age, sex, COPD severity (GOLD stage), and resting oxygen saturation (SpO_2_). Adjusted odds ratios (aORs) with 95% confidence intervals (CIs) and corresponding *p* values were reported. Given the sample size and outcome distribution, these multivariable analyses were considered exploratory. Sensitivity analyses additionally included smoking status, chronic ischemic heart disease, and beta-blocker use.

## 3. Results

A total of 100 patients with confirmed COPD were included in the analysis and stratified according to GOLD severity stages. Comprehensive clinical, inflammatory, oxidative stress, echocardiographic, and electrocardiographic parameters were evaluated to investigate the relationship between disease severity and cardiac remodeling. The results revealed progressive alterations in both cardiac structure and function across COPD stages, supporting the presence of a complex cardiopulmonary interaction driven by chronic inflammation and oxidative stress.

Baseline clinical characteristics are summarized in Table 1. The study population comprised 100 patients with COPD, including 55 patients with GOLD 1–2, 33 with GOLD 3, and 12 with GOLD 4 disease. Significant differences across GOLD severity groups were observed for BMI, smoking status, resting oxygen saturation, long-term oxygen therapy use, dyslipidemia, and inhaled corticosteroid use (all *p* < 0.05). Patients with GOLD 4 COPD exhibited the lowest resting SpO_2_ and the highest use of long-term oxygen therapy, consistent with more advanced respiratory impairment. Cardiovascular comorbidities were highly prevalent across the study population, particularly hypertension, heart failure, ischemic heart disease, and dyslipidemia.

### 3.1. Structural Cardiac Remodeling Assessed by Echocardiography

Echocardiographic parameters were compared across GOLD severity stages to assess structural cardiac remodeling in relation to COPD severity. Significant differences were identified in several parameters reflecting left-sided cardiac geometry, with the most pronounced changes observed in patients with GOLD 4 COPD. Left atrial volume differed significantly across GOLD categories (*p* = 0.016), with lower values in the GOLD 4 group compared with patients with less advanced disease. Left atrial height showed a similar pattern and was significantly reduced in GOLD 4 patients (*p* = 0.027). In addition, left atrial diameter tended to decrease with increasing COPD severity, although the difference did not reach statistical significance (*p* = 0.061), whereas left atrial width remained comparable across GOLD categories (*p* = 0.411).

Differences were also observed in left ventricular structural parameters. Left ventricular end-diastolic volume showed a tendency to decrease with increasing disease severity, with the lowest values observed in patients with GOLD 4 COPD, although the overall difference did not reach statistical significance (*p* = 0.093). Interventricular septal thickness during systole differed significantly across GOLD stages (*p* = 0.045), with lower values in the most advanced disease group. In contrast, interventricular septal thickness during diastole did not differ significantly according to COPD severity.

Despite these changes in cardiac chamber dimensions and myocardial geometry, left ventricular ejection fraction remained comparable across GOLD categories, with no significant differences according to disease severity. Overall, the echocardiographic analysis identified differences predominantly in structural parameters rather than in global left ventricular systolic function. Detailed numerical values and post hoc comparisons for the echocardiographic parameters according to GOLD stage are presented in Table 2.

#### 3.1.1. Secondary Exploratory Analysis of Sex-Related Differences in Cardiac Remodeling Parameters

Table 3 illustrates sex-specific differences in echocardiographic parameters. Left ventricular ejection fraction (EF) was significantly higher in women (66.66 ± 5.201%) compared to men (59.96 ± 11.527%, *p* = 0.021), suggesting better preservation of systolic function in female patients. Left ventricular posterior wall thickness in diastole (LVPWTd) and systole (LVPWTs) were both significantly greater in men (*p* = 0.039 and *p* = 0.006, respectively), indicating a more pronounced pattern of myocardial hypertrophy in the male subgroup. Left ventricular systolic mass (LV MASS s) was also significantly higher in men (247.29 ± 77.574 g vs. 204.71 ± 83.206 g, *p* = 0.018). Among diastolic parameters, late diastolic mitral inflow velocity (A Vel) was significantly higher in women (60.17 ± 4.325 cm/s vs. 55.26 ± 11.27 cm/s, *p* = 0.034), reflecting a greater dependence on atrial contraction for left ventricular filling. Aortic root diameter (AD) and left ventricular outflow tract diameter (LVOT diam) were significantly larger in men (*p* = 0.044 and *p* = 0.045, respectively), as was the aortic annulus diameter (*p* = 0.007).

### 3.2. Electrical Cardiac Remodeling Assessed by 24 h Monitoring

**Figure 3:**
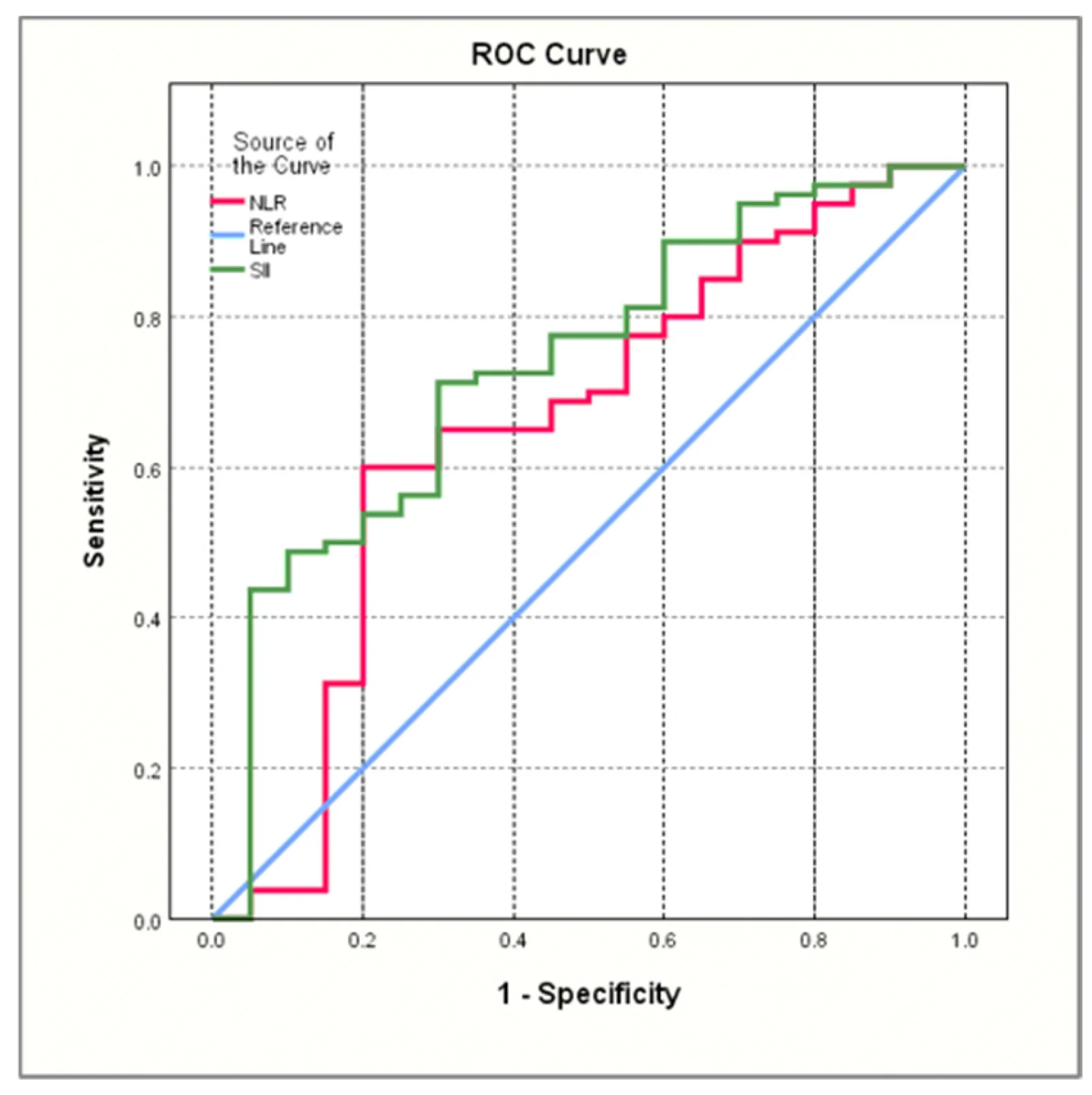
ROC analysis—sinus rhythm abnormalities.

Among patients stratified by the presence of sinus rhythm abnormalities on 24 h Holter monitoring, the systemic immune-inflammation index (SII) was significantly elevated in those with rhythm disturbances compared to those without (median 542.08 vs. 378.38, *p* = 0.001). The neutrophil-to-lymphocyte ratio (NLR) was also significantly higher in patients with sinus rhythm abnormalities (median 2.30 vs. 1.91, *p* = 0.033). In contrast, circulating cytokine levels (IL-6, IL-8, IL-1β, TNF-α) and classical inflammatory markers (CRP, ESR, fibrinogen) did not differ significantly between groups. ROC analysis identified SII as the best predictor of sinus rhythm abnormalities (AUC = 0.736, cut-off 443.52, sensitivity 71.3%, specificity 70.0%), followed by NLR (AUC = 0.654, cut-off 2.09, sensitivity 60.0%, specificity 80.0%) (Table 4 and Table 5, Figure 3).

**Figure 4:**
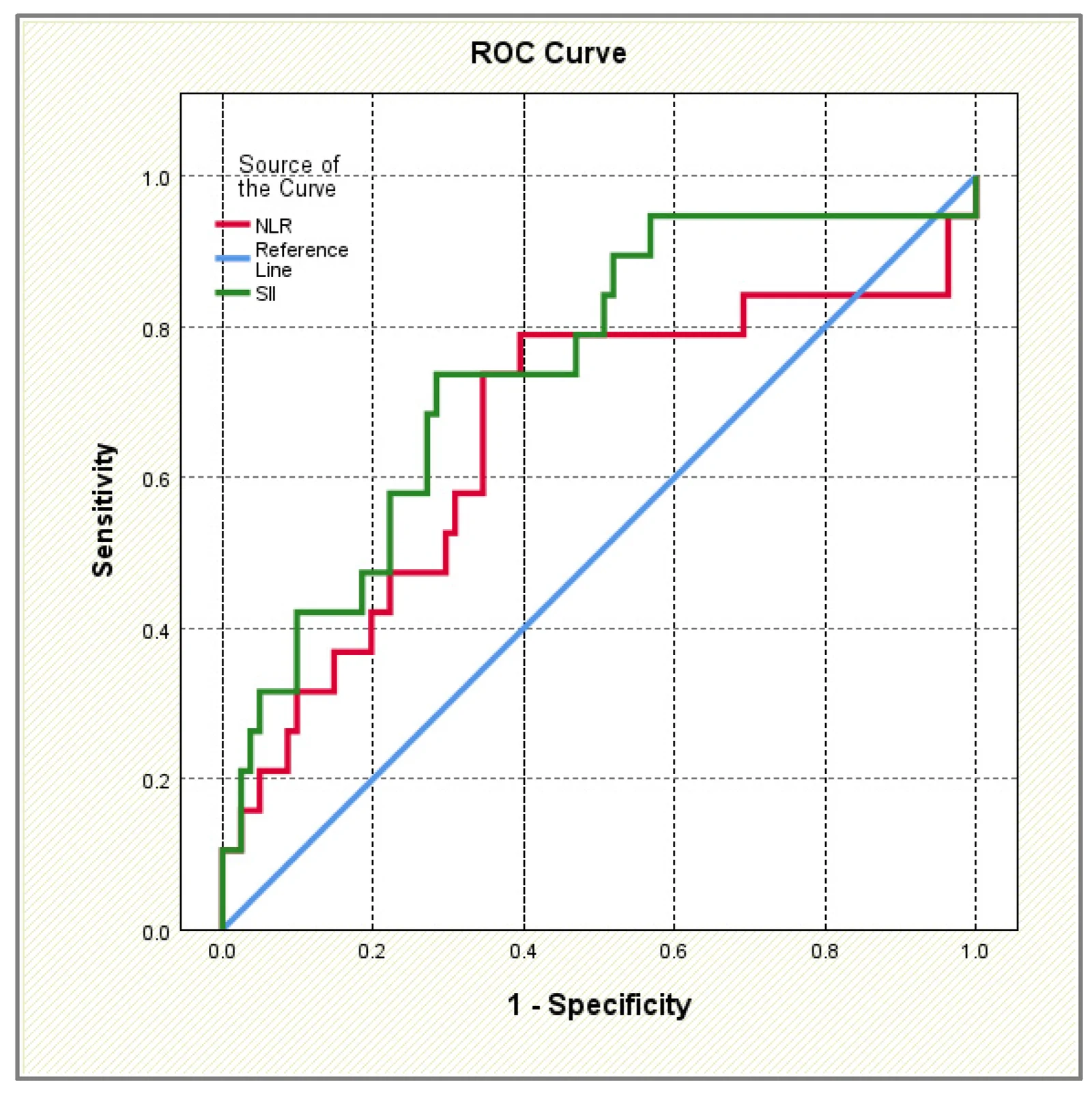
ROC analysis—atrial fibrillation/atrial flutter.

Patients without atrial fibrillation or atrial flutter demonstrated significantly higher SII values in this group compared to those with it (median 541.88 vs. 371.54, *p* < 0.001), indicating that higher SII characterized patients with preserved sinus rhythm who subsequently developed this arrhythmia—a pattern consistent with the role of systemic inflammation in atrial remodeling. NLR was significantly elevated in patients with atrial fibrillation/flutter (median 2.30 vs. 1.91, *p* = 0.032). Circulating cytokines and classical inflammatory markers did not reach statistical significance. ROC curve analysis demonstrated that low SII values were associated with the presence of atrial fibrillation/flutter (AUC = 0.745, cut-off 443.52, sensitivity 73.7%, specificity 71.6%), and low NLR showed similar discriminatory ability (AUC = 0.659, cut-off 2.06, sensitivity 78.9%, specificity 59.3%) (Table 6 and Table 7, Figure 4).

**Figure 5:**
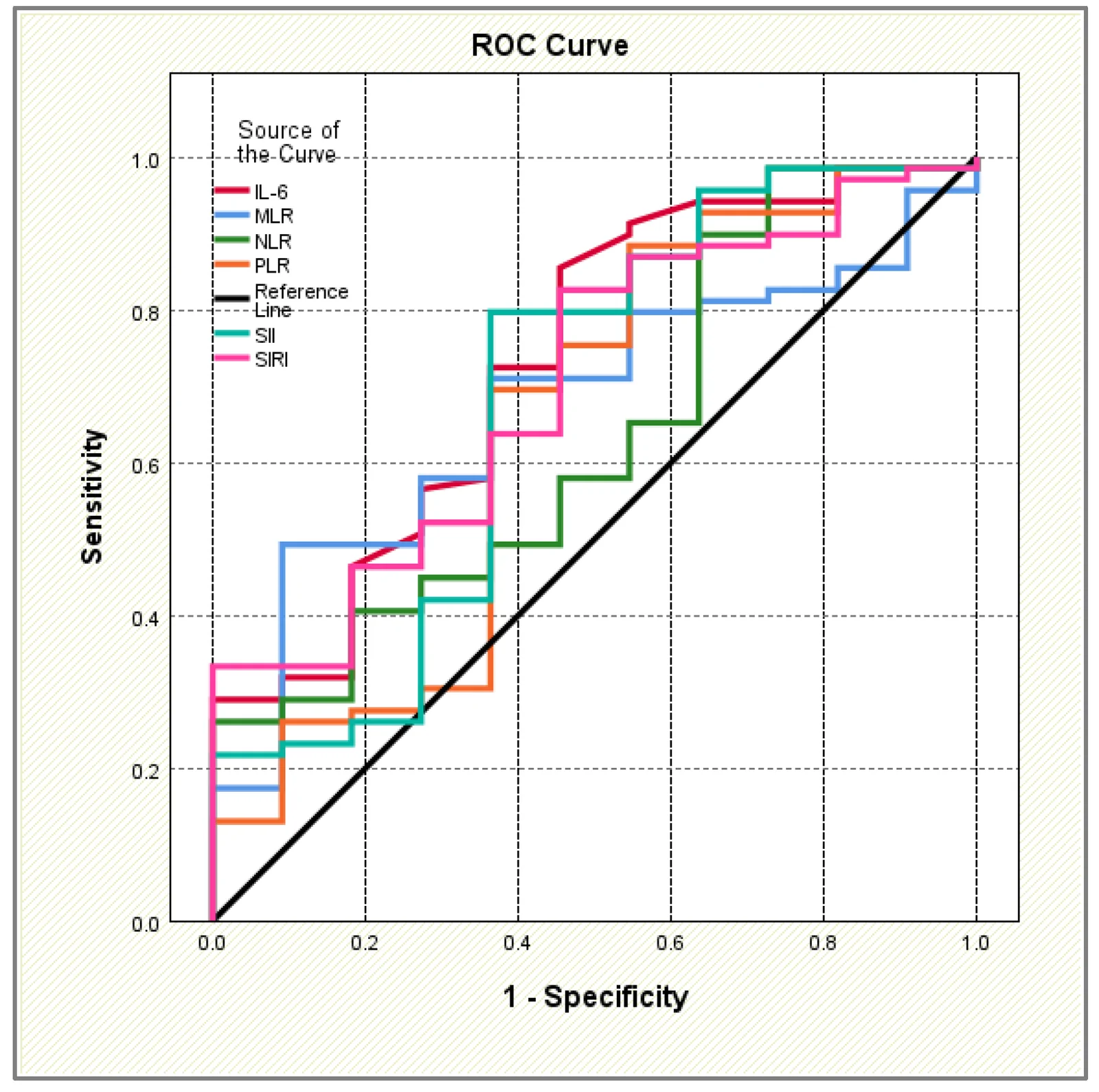
ROC analysis—VPCs.

Patients with ventricular ectopic beats (VPCs) exhibited a broader pattern of inflammatory activation compared to those with other arrhythmia types. IL-6 was significantly elevated in VPC-positive patients (median 40.85 vs. 25.69 pg/mL, *p* = 0.014). SII was highly significantly increased in patients with VPCs (median 538.72 vs. 358.25, *p* = 0.007), as were SIRI (*p* = 0.011), MLR (*p* = 0.026), NLR (*p* = 0.037), and PLR (*p* = 0.049). ROC analysis identified IL-6 as the strongest predictor (AUC = 0.732, cut-off 26.09 pg/mL, sensitivity 85.5%, specificity 54.5%), followed by SIRI (AUC = 0.702, sensitivity 82.6%, specificity 54.5%) and SII (AUC = 0.682). MLR demonstrated high specificity (91.9%) at a cut-off of 0.25 (Table 8 and Table 9, Figure 5).

**Figure 6:**
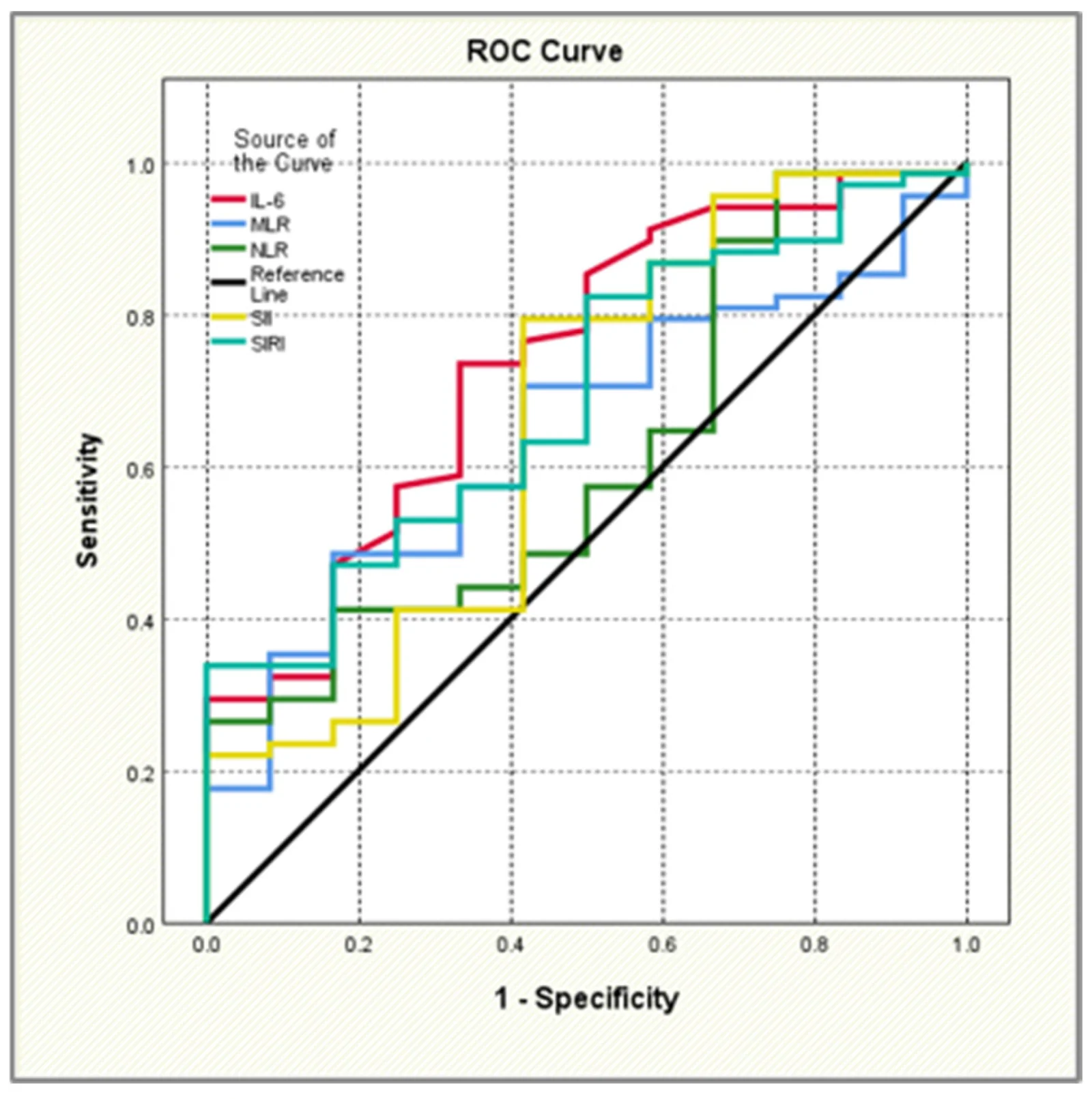
ROC analysis—isolated supraventricular ectopic beats.

The analysis restricted to isolated supraventricular premature complexes yielded results closely consistent with those observed for the broader SVPC category. IL-6 remained highly significantly elevated (*p* = 0.009), and all four composite inflammatory indices—SII (*p* = 0.002), SIRI (*p* = 0.005), MLR (*p* = 0.013), and NLR (*p* = 0.011)—were significantly higher in patients with isolated supraventricular ectopy. These findings reinforce the concept that even isolated supraventricular beats reflect a persistent pro-inflammatory state capable of promoting atrial electrical instability (Table 10 and Table 11, Figure 6).

In patients presenting with supraventricular couplets, SIRI (*p* = 0.015) and MLR (*p* = 0.023) were the only inflammatory indices to reach statistical significance, being significantly higher in those with this arrhythmia pattern. Other composite indices (SII, NLR, PLR) and all circulating cytokines did not differ significantly between groups, suggesting that supraventricular couplets may be associated with monocyte-driven inflammatory activation rather than a broad innate immune response. Fibrinogen showed a borderline trend (*p* = 0.054) toward higher values in patients with couplets (Table 12).

**Figure 7:**
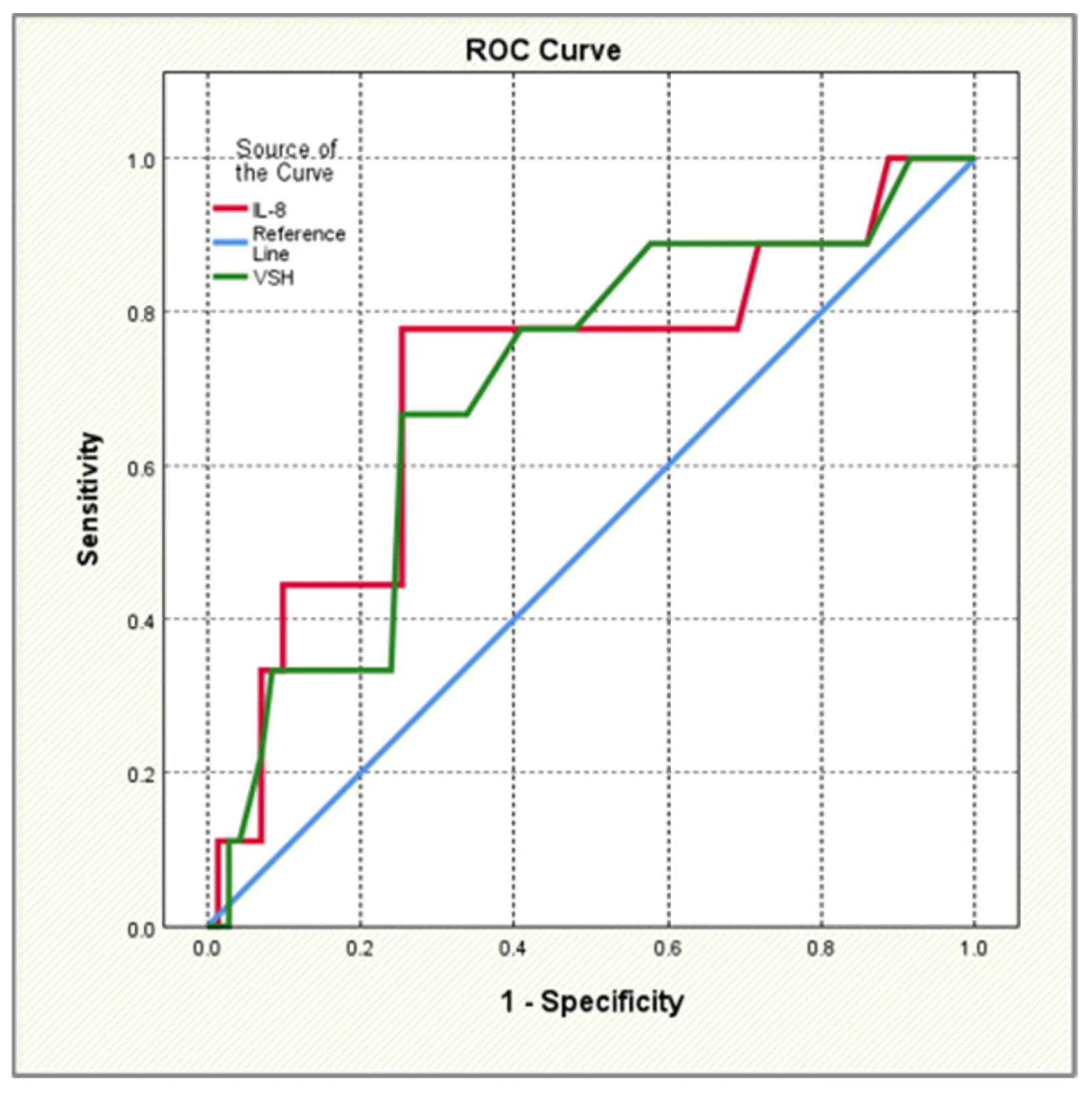
ROC analysis—PSVT.

Among patients with paroxysmal supraventricular tachycardia (PSVT), IL-8 was significantly elevated (median 160.09 vs. 91.34 pg/mL, *p* = 0.039), identifying neutrophil-driven inflammatory signaling as a potential contributor to this arrhythmia. ESR (erythrocyte sedimentation rate) was also significantly higher in PSVT-positive patients (median 20.00 vs. 12.50 mm/h, *p* = 0.035). Composite inflammatory indices and other cytokines did not reach statistical significance, though SIRI showed a borderline trend (*p* = 0.066). ROC analysis showed that IL-8 had the highest discriminatory performance for PSVT (AUC = 0.712, cut-off 149.84 pg/mL, sensitivity 77.8%, specificity 74.6%), followed by ESR (AUC = 0.701, cut-off 18.50 mm/h, sensitivity 66.7%, specificity 74.6%) (Table 13 and Table 14, Figure 7).

**Figure 8:**
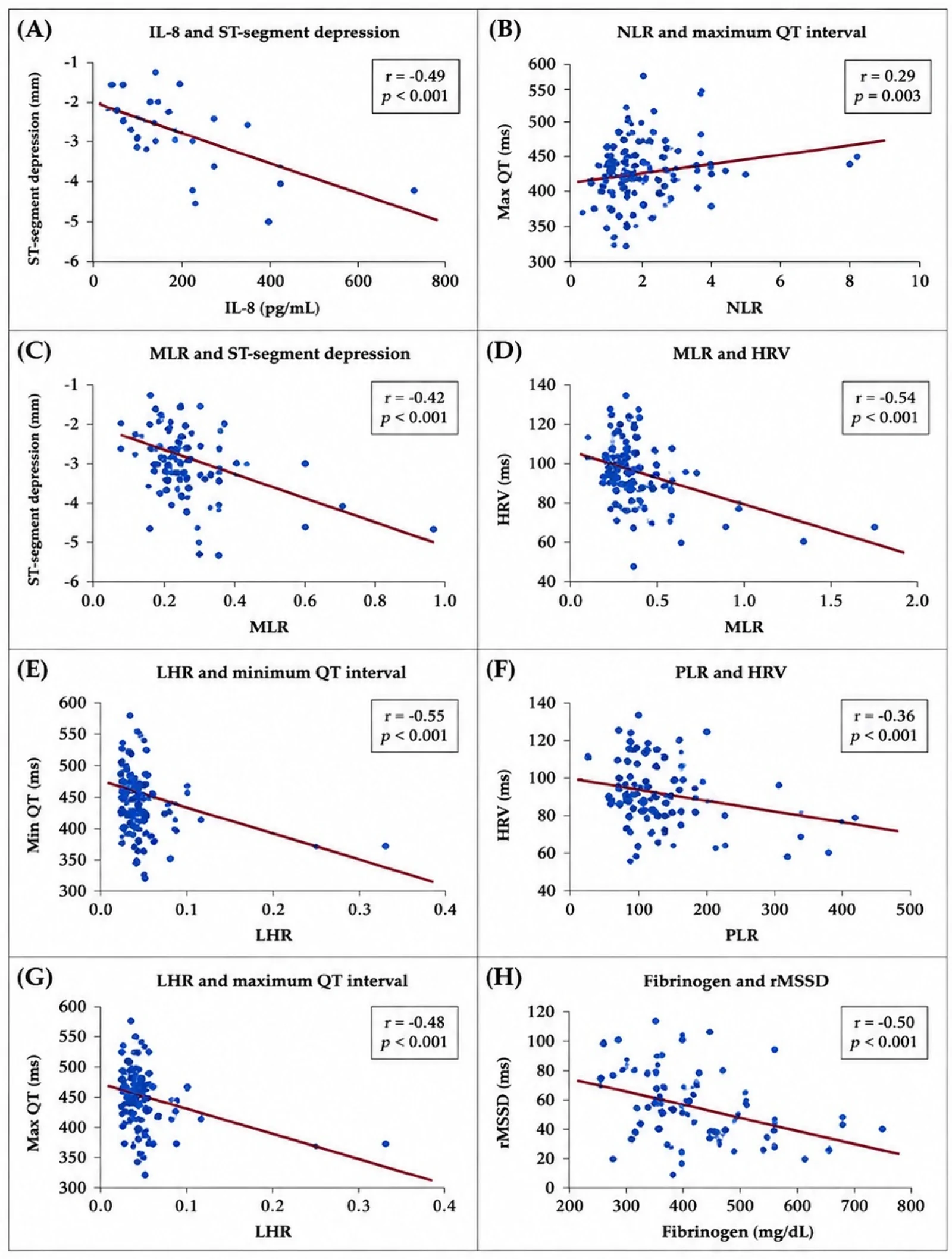
Correlations between inflammatory biomarkers and electrocardiographic parameters. (**A**) IL-8 and ST-segment depression; (**B**) NLR and maximum QT interval; (**C**) MLR and ST-segment depression; (**D**) MLR and HRV; (**E**) LHR and minimum QT interval; (**F**) PLR and HRV; (**G**) LHR and maximum QT interval; and (**H**) fibrinogen and rMSSD. Regression lines illustrate the direction of the observed associations. Abbreviations: NLR, neutrophil-to-lymphocyte ratio; MLR, monocyte-to-lymphocyte ratio; LHR, lymphocyte-to-HDL ratio; PLR, platelet-to-lymphocyte ratio; HRV, heart rate variability; rMSSD, root mean square of successive differences.

Table 15 summarizes the significant correlations identified between inflammatory biomarkers and electrocardiographic parameters. IL-8 showed a statistically significant moderate inverse correlation with ST-segment depression (r = −0.550, *p* = 0.041), suggesting that higher IL-8 concentrations are associated with more pronounced ischemic repolarization abnormalities. MLR demonstrated a significant inverse correlation with ST-segment depression (r = −0.481, *p* = 0.037) and a highly significant inverse correlation with heart rate variability (HRV) (r = −0.297, *p* = 0.007). NLR was significantly and positively correlated with maximum QT interval (r = 0.222, *p* = 0.046) and maximum corrected QT interval (QTc) (r = 0.235, *p* = 0.035), indicating that higher neutrophil-to-lymphocyte ratios are associated with prolonged ventricular repolarization. LHR showed significant inverse correlations with both maximum QT (r = −0.246, *p* = 0.027) and QTc (r = −0.242, *p* = 0.029). PLR was significantly and inversely correlated with HRV (r = −0.228, *p* = 0.041). Fibrinogen demonstrated a significant inverse correlation with rMSSD (r = −0.433, *p* = 0.019), reflecting the close interaction between coagulation-related inflammation and impaired parasympathetic autonomic modulation (Figure 8).

### 3.3. Exploratory Multivariable Analysis

To assess whether the observed associations between systemic inflammatory indices and electrical cardiac abnormalities persisted after accounting for clinically relevant potential confounders, exploratory multivariable logistic regression analyses were performed. After adjustment for age, sex, COPD severity (GOLD stage), and resting SpO_2_, both SII and NLR remained significantly associated with sinus rhythm abnormalities (Table 16). Sensitivity analyses additionally accounting for smoking status, chronic ischemic heart disease, and beta-blocker use yielded comparable estimates.

Given the sample size and outcome distribution, these multivariable findings should be interpreted as exploratory and require confirmation in larger independent cohorts.

## 4. Discussion

The present study provides an integrated assessment of structural and electrical cardiac abnormalities in patients with COPD in relation to systemic inflammatory biomarkers. The main findings were: (1) COPD severity was associated with significant differences in left atrial and selected left ventricular structural parameters despite preserved left ventricular ejection fraction; (2) systemic inflammatory indices, particularly SII and NLR, were associated with electrical abnormalities detected by 24 h Holter monitoring; (3) specific inflammatory markers showed associations with selected arrhythmic phenotypes, including IL-6 with ventricular and supraventricular ectopic activity and IL-8 with PSVT and ST-segment depression; and (4) several inflammatory indices were associated with measures of ventricular repolarization and autonomic function. These findings support an association between systemic inflammatory activity and cardiovascular involvement in COPD, while the cross-sectional design does not allow causal or temporal relationships to be established.

Structural cardiac remodeling was evident across COPD severity stages, particularly through changes in left atrial geometry and selected left ventricular parameters, while left ventricular ejection fraction remained preserved. These findings are consistent with the concept that cardiovascular involvement may occur in COPD before overt global systolic dysfunction becomes apparent [24,25]. Previous experimental and clinical studies have proposed oxidative stress, systemic inflammation, hypoxemia, and mitochondrial dysfunction as potential contributors to cardiovascular remodeling in COPD [26,27,28,29,30]. Importantly, these mechanisms were not directly investigated in the present study and should therefore be regarded as biologically plausible explanations for the observed associations rather than mechanisms demonstrated by our data.

Patients with GOLD stage 4 COPD exhibited smaller left atrial dimensions, while left ventricular ejection fraction remained preserved. This pattern is consistent with previous studies indicating that pulmonary hyperinflation and impaired ventricular filling may reduce cardiac preload and chamber dimensions in advanced COPD [31,32,33,34]. Increased intrathoracic pressure, reduced pulmonary venous return, and ventricular interdependence have been proposed as mechanisms contributing to impaired left ventricular filling and smaller cardiac chamber dimensions in severe COPD [31,32,33]. Thus, the reduced atrial dimensions observed in advanced disease may reflect altered cardiopulmonary mechanics and impaired filling rather than the absence of myocardial remodeling.

The preservation of left ventricular ejection fraction despite structural differences is consistent with previous echocardiographic studies showing that subclinical cardiac abnormalities may occur in COPD while conventional systolic function remains preserved [34].

Within the cardiopulmonary continuum, previous studies have proposed systemic inflammation and oxidative stress as potential pathways linking pulmonary disease to cardiovascular remodeling [35,36,37]. Inflammatory mediators evaluated in our cohort, including IL-6, IL-8, and IL-1β, have been implicated in endothelial dysfunction, oxidative injury, myocardial fibrosis, and extracellular matrix remodeling [38,39,40], while composite indices such as NLR and PLR may reflect a broader systemic pro-inflammatory state [41,42]. Although these mechanisms were not directly investigated in the present study, they provide biological plausibility for the observed cardiac abnormalities and support further evaluation of inflammatory biomarkers alongside echocardiographic parameters in multimodal cardiovascular risk assessment.

Further evidence of cardiac remodeling emerged from the assessment of ventricular geometry, with significant differences in IVSTs, LVIDs, and LVOT diameter across GOLD stages, despite comparable IVSTd. These structural differences may reflect the combined effects of pulmonary and systemic factors associated with COPD. In particular, the reduced IVSTs and LVIDs observed in GOLD stage 4 may be partly related to progressive pulmonary hyperinflation, which can increase intrathoracic pressure and impair ventricular filling [43].

Additional differences in IVSTs, LVIDs, and LVOT diameter further suggest that COPD severity is associated with alterations in ventricular geometry. Previous studies have related similar changes to pulmonary hyperinflation and impaired ventricular filling, while oxidative stress and systemic inflammation have been proposed as additional contributors to myocardial and vascular remodeling [44,45,46,47]. The reduced LVOT diameter observed in severe COPD may reflect diminished preload and ventricular filling, consistent with previous reports of reduced left ventricular end-diastolic volume and cardiac output in severe emphysema [48,49,50].

Right atrial and right ventricular dimensions did not significantly differ across GOLD stages, suggesting that overt chamber dilatation was not a prominent feature in our cohort. As functional impairment may precede structural enlargement, complementary measures such as TAPSE, RV strain, and pulmonary artery systolic pressure may provide additional information. Overall, our findings indicate a pattern of altered cardiac geometry without overt deterioration of left ventricular systolic function [51]. Previous literature suggests that pulmonary hyperinflation, systemic inflammation and oxidative stress, autonomic dysregulation, and ventricular interdependence may contribute to this complex cardiopulmonary interaction [52].

Sex-specific analysis revealed differences in cardiac remodeling despite the absence of significant overall differences in left atrial dimensions. Men showed greater left ventricular wall thickness and mass, larger aortic and LVOT dimensions, and greater left atrial anteroposterior diameter, whereas women exhibited higher left ventricular ejection fraction. These findings are consistent with previously described sex-related differences in cardiovascular adaptation, characterized by greater myocardial mass and wall thickness in men and relatively preserved systolic function in women [53,54,55].

Another noteworthy finding was the significantly higher A-wave velocity in women, suggesting a greater dependence on atrial contraction for left ventricular filling. This observation may indicate early impairment of left ventricular relaxation despite preserved ejection fraction and may represent one of the earliest manifestations of diastolic dysfunction [56]. Previous studies have implicated oxidative stress and chronic inflammation in myocardial stiffening and extracellular matrix remodeling, providing a potential biological explanation for subclinical diastolic abnormalities preceding overt systolic dysfunction [57]. Taken together, these findings suggest sex-related differences in the pattern of cardiac remodeling in COPD, with more pronounced structural changes in men and relatively preserved systolic function in women, despite potential alterations in ventricular compliance [58]. These findings support further investigation of sex-specific differences in cardiovascular remodeling in COPD.

The echocardiographic findings observed in our cohort may be interpreted within the framework of oxidative stress and systemic inflammation as potential pathways linking pulmonary and cardiovascular remodeling. Although these mechanisms were not directly investigated in our study, previous evidence supports their biological plausibility [59]. Cigarette smoke exposure, chronic hypoxemia, systemic inflammation, and mitochondrial dysfunction have been associated with increased ROS production and impaired antioxidant defenses [19], potentially activating redox-sensitive pathways involved in endothelial dysfunction, myocardial fibrosis, extracellular matrix remodeling, and autonomic imbalance [60].

Within this framework, the inflammatory biomarkers evaluated in our cohort may reflect different components of a broader pro-inflammatory and pro-oxidative milieu potentially associated with electrical and structural myocardial remodeling [61]. However, our findings demonstrate associations rather than direct mechanistic relationships. This interpretation is consistent with previous imaging studies showing impaired left ventricular filling and reduced ventricular dimensions in COPD [62,63], while mitochondrial dysfunction, redox signaling, inflammation, and fibrosis have been proposed as potential biological mechanisms underlying COPD-related systemic and cardiovascular alterations [64]. Together, these observations support further evaluation of inflammatory biomarkers alongside echocardiography and rhythm monitoring within a multimodal cardiovascular assessment of COPD.

Electrical remodeling was associated with higher SII and NLR, whereas circulating IL-6, IL-8, IL-1β, and TNF-α did not significantly differ between groups. These findings suggest that composite inflammatory indices may better reflect the systemic inflammatory burden associated with electrical abnormalities than isolated cytokine measurements. By integrating multiple immune cell populations, SII and NLR may provide a more stable representation of persistent systemic inflammation than individual cytokines, which are characterized by greater biological variability and shorter plasma half-lives [65,66].

The associations of SII and NLR with electrical abnormalities are biologically plausible based on previous evidence. Neutrophil activation has been linked to increased ROS production, ion-channel and connexin dysfunction, altered calcium handling, and myocardial fibrosis, all of which may contribute to electrical instability [67]. Similarly, elevated NLR reflects an imbalance between innate inflammatory activation and adaptive immune regulation, with neutrophil-mediated oxidative injury and lymphopenia potentially contributing to endothelial and myocardial dysfunction [68]. These mechanisms, although not directly assessed in our study, may provide a biological explanation for the observed associations.

Our findings are consistent with previous studies linking systemic inflammation to atrial remodeling and atrial fibrillation [69]. Hu et al. [70] reported that elevated NLR independently predicted atrial fibrillation recurrence after catheter ablation, supporting its association with electrical abnormalities across different cardiovascular settings. Similarly, SII has been associated with adverse cardiovascular outcomes and mortality in chronic heart failure and atrial fibrillation populations [71,72], supporting its potential value as an integrated marker of systemic inflammatory burden. In contrast, circulating cytokines did not significantly differ between groups, possibly reflecting their biological variability, episodic secretion, and rapid turnover. Composite cellular indices such as SII and NLR may therefore provide a more stable representation of persistent systemic inflammatory activity, although their relationship with cardiac electrical abnormalities requires prospective validation.

Collectively, our findings suggest that electrical cardiac abnormalities in COPD are associated with persistent systemic inflammatory activity rather than isolated cytokine elevations [73]. SII and NLR showed moderate discriminatory performance in ROC analyses and may provide complementary information regarding cardiovascular involvement; however, the observed AUC values do not support their use as standalone diagnostic or prognostic tools. The higher discriminatory performance of SII may reflect its integration of neutrophil, lymphocyte, and platelet counts, providing a broader estimate of systemic immune-inflammatory activity than individual cytokine measurements [74]. These findings should be interpreted cautiously, and the potential clinical utility of these indices requires validation in prospective, independent cohorts.

Our ROC findings are consistent with previous studies associating elevated SII with adverse cardiovascular outcomes, mortality, and atrial fibrillation [70,71,75], while NLR has been identified as a predictor of atrial fibrillation recurrence and electrical instability in different cardiovascular populations [70,76]. Together, these observations support the potential relevance of composite inflammatory indices as markers of systemic inflammatory activity associated with cardiac electrical abnormalities. From a biological perspective, SII and NLR may reflect interconnected inflammatory pathways potentially involved in electrical remodeling; however, these mechanisms were not directly assessed in the present study. Libby et al. [75] and Hansson et al. [76] proposed that chronic inflammation promotes endothelial dysfunction and thrombogenicity through sustained immune activation, while Steven, Münzel, and Daiber [77,78] emphasized the pivotal role of oxidative stress in coupling inflammation with cardiovascular remodeling. Together, these mechanisms provide a plausible biological explanation for the predictive performance of SII observed in the present study.

From a clinical perspective, SII and NLR may provide complementary information regarding cardiovascular involvement in COPD; however, their moderate discriminatory performance does not support their use as standalone screening, diagnostic, or prognostic tools. Their potential value may lie in future multimodal risk models integrating inflammatory, echocardiographic, and electrocardiographic parameters, which require prospective validation.

The moderate performance observed in our study also suggests that electrical cardiac abnormalities in COPD are likely multifactorial, potentially involving hypoxemia, autonomic dysfunction, pulmonary vascular changes, ventricular interdependence, mitochondrial dysfunction, and oxidative stress. Accordingly, inflammatory indices should be regarded as complementary markers. The lower discriminatory performance of NLR compared with SII may partly reflect its narrower representation of systemic inflammation, as it does not incorporate platelet-related inflammatory and thrombo-inflammatory activity.

Our findings are consistent with previous studies supporting the prognostic relevance of composite inflammatory indices in cardiovascular disease. SII has been associated with long-term cardiovascular mortality [71], while NLR has been linked to atrial fibrillation recurrence, atrial structural remodeling, and myocardial fibrosis [70]. Composite indices may better reflect persistent systemic inflammatory activity by integrating multiple immune and thrombo-inflammatory components potentially related to oxidative stress and cardiovascular remodeling [79]. However, the moderate AUC values observed in our cohort, particularly for NLR, indicate that these biomarkers provide complementary rather than standalone information regarding cardiac electrical abnormalities in COPD.

Patients with atrial fibrillation or atrial flutter exhibited significantly higher SII and NLR, whereas circulating IL-6, IL-8, IL-1β, and TNF-α did not significantly differ between groups. This pattern suggests that composite inflammatory indices may better reflect the systemic inflammatory activity associated with atrial arrhythmias than individual cytokine measurements. Previous studies have implicated platelet-mediated inflammatory and profibrotic pathways in atrial remodeling [80,81], while oxidative stress has been proposed to contribute to ion-channel dysfunction, altered calcium handling, connexin remodeling, and myocardial fibrosis [82]. These mechanisms provide biological plausibility for the observed associations but were not directly assessed in the present study.

The ROC analysis showed higher discriminatory performance of SII compared with NLR for atrial fibrillation/flutter, with an AUC of 0.736. This finding suggests that an index integrating multiple immune cell populations may provide broader information on systemic inflammatory activity associated with atrial arrhythmias. However, the moderate discriminatory ability does not support the use of SII as a standalone screening, diagnostic, or prognostic tool. Given its low cost and routine availability, SII may provide complementary information alongside clinical, electrocardiographic, and echocardiographic assessment, although its potential clinical value requires prospective validation.

Unlike atrial fibrillation/flutter, ventricular premature complexes were associated with higher circulating IL-6 levels, while IL-8 showed a non-significant trend. This finding suggests a potential association between ventricular ectopy and inflammatory activity. Previous evidence has implicated IL-6 in myocardial inflammatory and profibrotic pathways [83], including fibroblast activation and extracellular matrix remodeling through JAK/STAT3 signaling [84]. These mechanisms may provide biological plausibility for the observed association but were not directly investigated in the present study [85].

Patients with ventricular premature complexes showed significantly higher SII, SIRI, MLR, NLR, and PLR values, together with elevated IL-6 concentrations, indicating an association between ventricular ectopy and a broader systemic inflammatory profile. Among the evaluated biomarkers, SII showed the strongest statistical association. Previous studies have implicated platelet-mediated inflammatory and profibrotic pathways in fibroblast activation and extracellular matrix remodeling [84,86], providing biological plausibility for the association between SII and ventricular ectopy. However, these mechanisms were not directly assessed in the present study.

Monocyte-related indices, including SIRI and MLR, were also significantly associated with ventricular ectopy, suggesting a potential contribution of monocyte-related inflammatory activity. Previous experimental evidence has implicated monocytes and macrophages in myocardial inflammation, fibrosis, and electrophysiological alterations through cytokine release and modulation of cardiomyocyte and gap-junction function [87,88,89]. These mechanisms may provide biological plausibility for the observed associations but were not directly assessed in our study.

SIRI showed greater discriminatory performance than NLR, potentially reflecting its integration of neutrophil, monocyte, and lymphocyte counts and thus a broader inflammatory profile [90]. This finding is consistent with previous literature implicating innate immune activation and oxidative stress in the cardiopulmonary continuum. Miklós et al. [91] described oxidative stress as a potential biological link between COPD and cardiovascular disease, involving endothelial dysfunction, vascular remodeling, and myocardial injury. These mechanisms may provide biological context for our findings but were not directly assessed in the present study.

In patients with supraventricular ectopic activity, IL-6 showed the highest discriminatory performance, while SIRI, SII, MLR, and NLR also demonstrated moderate discrimination. Previous studies have implicated inflammatory cytokines, particularly IL-6, in atrial electrophysiological alterations through effects on ion-channel and connexin function [92,93]. Similarly, SIRI has been associated with adverse cardiovascular outcomes and has been proposed as a marker of chronic innate immune activation [94,95,96]. These mechanisms provide biological plausibility for the observed associations but were not directly assessed in our study. Given the exploratory nature of these subgroup analyses, the findings should be interpreted cautiously and require confirmation in larger prospective cohorts.

Correlation analyses identified significant associations between inflammatory biomarkers and electrocardiographic parameters. The strongest association was observed between IL-8 and ST-segment depression (r = −0.550, *p* = 0.041), suggesting a relationship between higher IL-8 levels and repolarization abnormalities. Previous evidence has implicated IL-8-mediated neutrophilic inflammation in oxidative stress, endothelial dysfunction, and microvascular injury [97,98], providing a potential biological explanation for this association. However, these mechanisms were not directly assessed in the present study [97]. Previous evidence has linked IL-8-mediated neutrophilic inflammation to oxidative stress, endothelial dysfunction, and microvascular injury, which may provide a biological explanation for the observed association with ST-segment abnormalities [98].

NLR showed weak positive correlations with both maximal QT interval (r = 0.222, *p* = 0.046) and QTc (r = 0.235, *p* = 0.035), suggesting an association between systemic inflammatory activity and ventricular repolarization. QT/QTc prolongation is associated with increased repolarization heterogeneity and arrhythmic risk [99]. Previous experimental evidence suggests that inflammatory signaling may influence cardiac ion-channel function and action potential duration, providing a potential biological explanation for these associations [100].

A particularly important finding was the significant inverse association between fibrinogen and rMSSD, highlighting a potential interaction between inflammation, coagulation activation, endothelial dysfunction, and impaired parasympathetic modulation [101,102,103]. This observation is consistent with previous evidence suggesting that coagulation-related inflammatory pathways may be associated not only with vascular injury but also with autonomic imbalance and cardiovascular risk in COPD. Taken together, these findings indicate that inflammatory biomarkers are associated with multiple components of cardiac involvement, including atrial ectopy, ST-segment abnormalities, ventricular repolarization abnormalities, and autonomic dysfunction. Previous evidence has proposed oxidative stress as a potential biological pathway linking chronic pulmonary inflammation with endothelial dysfunction, mitochondrial injury, autonomic imbalance, and myocardial fibrosis [19]. Although this framework provides biological plausibility for our observations, oxidative stress was not directly assessed in the present study, and therefore its mechanistic role cannot be established from our findings.

### Limitations

Several limitations should be acknowledged. First, the single-center, cross-sectional design and relatively modest sample size limit generalizability and preclude conclusions regarding causality or temporal relationships. In addition, the findings were not externally validated in an independent cohort. Second, some analyses involved relatively small arrhythmic subgroups, resulting in wide confidence intervals and limited statistical precision; these findings should therefore be considered preliminary and hypothesis-generating. Third, although exploratory multivariable models accounted for several clinically relevant confounders, including age, sex, GOLD stage, oxygenation status, smoking status, chronic ischemic heart disease, and beta-blocker use, residual confounding from unmeasured or incompletely characterized factors cannot be excluded. Furthermore, concomitant respiratory and cardiovascular therapies may have influenced both inflammatory biomarker levels and arrhythmic burden. Although beta-blocker use was considered in sensitivity analyses, the effects of individual medication classes could not be comprehensively assessed, and residual pharmacological confounding cannot be excluded.

In addition, multiple exploratory statistical analyses were performed across inflammatory biomarkers, arrhythmic phenotypes, ROC curves, and correlation analyses. Although post hoc pairwise comparisons following significant omnibus tests were adjusted for multiple comparisons, no global correction was applied across the entire set of exploratory analyses, increasing the possibility of type I error and false-positive associations. Consequently, statistically significant findings should be interpreted as hypothesis-generating rather than confirmatory. Finally, inflammatory biomarkers were measured at a single time point, and the study did not directly assess oxidative stress pathways or the molecular mechanisms proposed to explain the observed associations. Prospective, longitudinal, multicenter studies with larger samples, external validation, and predefined cardiovascular endpoints are needed to validate these findings and determine their potential clinical relevance.

## 5. Conclusions

This study demonstrates significant associations between systemic inflammatory markers and structural and electrical cardiac abnormalities in patients with COPD. Echocardiographic changes across GOLD severity stages were predominantly related to cardiac geometry, particularly left atrial and selected left ventricular parameters, while left ventricular systolic function remained preserved. These findings highlight the presence of cardiovascular alterations in COPD even in the absence of overt systolic dysfunction.

Among the inflammatory biomarkers evaluated, IL-6 showed the highest discriminatory performance for selected supraventricular electrical abnormalities, whereas IL-8 was associated with transient ST-segment depression and paroxysmal supraventricular tachycardia. Composite inflammatory indices, particularly SII and NLR, were also associated with parameters of electrical remodeling. Taken together, these findings suggest that systemic inflammatory markers may provide complementary information regarding cardiovascular involvement in COPD, although their moderate discriminatory performance does not support their use as standalone diagnostic or prognostic tools.

The associations observed between inflammatory biomarkers, echocardiographic changes, and Holter-derived electrical abnormalities are consistent with a complex interplay between systemic inflammation, oxidative stress-related pathways, and cardiovascular involvement in COPD. However, the cross-sectional design of the study precludes conclusions regarding causality or temporal relationships. Accordingly, the identified associations and ROC-derived cutoff values should be considered exploratory and interpreted within the context of the present cohort.

Prospective, longitudinal, and multicenter studies are needed to validate these findings and determine whether combining inflammatory biomarkers with echocardiographic and ambulatory electrocardiographic assessment could contribute to improved cardiovascular risk stratification in COPD.

## Figures and Tables

**Table 1:** Baseline clinical characteristics of the study population according to COPD severity.

Characteristics	Total (n = 100)	GOLD 1-2 (n = 55)	GOLD 3 (n = 33)	GOLD 4 (n = 12)	*p*-Value
Age, years	67.02 ± 9.30	66.38 ± 10.68	68.45 ± 5.85	66.00 ± 10.51	0.812
Male sex, n (%)	68 (68%)	31 (56.4%)	26 (78.8%)	11 (91.7%)	0.011 *
BMI, kg/m^2^	30.55 ± 6.66	30.82 ± 6.03	32.30 ± 7.09	24.48 ± 4.96	**0.003** *
**Smoking status**					0.026 *
Current smoker, n (%)	25 (25%)	16 (29.1%)	7 (21.2%)	2 (16.7%)	
Former smoker, n	49 (49.0%)	20 (36.4%)	19 (57.6%)	10 (83.3%)	
Never smoker, n	26 (26%)	19 (34.5%)	7 (21.1%)	0 (0.0%)	
Pack-years	22.64 ± 20.24	18.53 ± 17.68	26.55 ± 23.33	30.75 ± 19.20	0.072
**Oxygenation parameters**					
Resting SpO_2_ on room air, %	93.73 ± 2.62	94.67 ± 1.48	93.27 ± 2.97	90.67 ± 3.14	**<0.001** *
Long-term/home oxygen use	12 (12%)	0 (0.0%)	0 (0.0%)	12 (100.0%)	**<0.001** *
**Cardiovascular comorbidities**					
Hypertension	85 (85.0%)	48 (87.3%)	29 (87.9%)	8 66.7%)	0.165
Chronic ischemic heart disease	56 (56%)	26 (47.3%)	20 (60.6%)	10 (83.3%)	0.060
Heart failure	63 (63.0%)	30 (54.5%)	23 (69.7%)	10 (83.3%)	0.108
Atrial fibrillation/flutter	19 (19.0%)	11 (20.0%)	8 (24.2%)	0 (0.0%)	0.179
Dyslipidemia	71 (71.0%)	39 (70.9%)	28 (84.8%)	4 (33.3%)	**0.003** *
Peripheral vascular disease	27 (27.0%)	14 (25.5%)	9 (27.3%)	4 (33.3%)	0.872
**Concomitant therapy**					
Short-acting bronchodilator	89 (89.0%)	48 (87.3%)	30 (90.9%)	11 (91.7%)	0.828
Long-acting bronchodilator	89 (89%)	48 (87.3%)	29 (87.9%)	12 (100.0%)	0.429
Inhaled corticosteroid	4 3 (43.0%)	16 (29.6%)	17 (58.6%)	10 (83.3%)	**<0.001** *
Blood pressure-lowering drugs	78 (78.0%)	44 (80.0%)	24 (72.7%)	10 (83.3%)	0.650
Beta-blockers	48 (48.0%)	26 (47.3%)	17 (51.5%)	5 (41.7%)	0.832
Statins	56 (56%)	28 (52.8%)	20 (69.0%)	5 (41.7%)	0.202
Other lipid-lowering drugs	25 (25.0%)	12 (21.8%)	12 (36.4%)	1 (8.3%)	0.114

Data are presented as mean ± standard deviation (SD) or frequency (%). Percentages were calculated within each COPD severity group. Comparisons among GOLD groups were performed using one-way ANOVA or Kruskal–Wallis test for continuous variables, as appropriate, and Pearson’s chi-squared test or Fisher’s exact test for categorical variables. * *p* < 0.05 was considered statistically significant.

**Table 2:** Echocardiographic characteristics of the study population according to GOLD stage.

Characteristics	Total (n = 100) Mean ± SD Median (IQR)	GOLD 1–2 (n = 55) Mean ± SD Median (IQR)	GOLD 3 (n = 33) Mean ± SD Median (IQR)	GOLD 4 (n = 12) Mean ± SD Median (IQR)	*p* Value
LA Vol (mL)	36.07 ± 14.444 34.6 (27.03 ÷ 45.6)	37.30 ± 14.348 37.2 (27.7 ÷ 49.6)	38.86 ± 12.365 37.7 (28.98 ÷ 45.38)	21.04 ± 14.259 17.5 (10.6 ÷ 27.7)	0.016 *
	Statistically significant differences between: GOLD stages 2 vs. 4 (*p* = 0.024); GOLD stages 3 vs. 4 (*p* = 0.016)				
LA H (mm)	46.72 ± 7.548 47 (40.95 ÷ 52.35)	47.47 ± 7.54 47.3 (41.5 ÷ 52.7)	47.81 ± 7.034 48.55 (42.68 ÷ 52.68)	39.61 ± 6.218 40.8 (33.3 ÷ 46.4)	0.027 *
	Statistically significant differences ‡ between: GOLD stages 2 vs. 4 (*p* = 0.031); GOLD stages 3 vs. 4 (*p* = 0.028)				
LA W (mm)	38.46 ± 6.17 38.25 (33.85 ÷ 42.58)	38.54 ± 6.29 38.4 (33.9 ÷ 42.5)	39.18 ± 4.92 38.45 (35.2 ÷ 43.33)	35.63 ± 9.216 32.9 (28.9 ÷ 40.3)	0.411
LA D (mm)	35.29 ± 8.212 34 (28.95 ÷ 38.2)	36.47 ± 8.734 36.5 (30.7 ÷ 38.2)	35.74 ± 7.307 33.45 (30.98 ÷ 39.4)	28.51 ± 6.164 27.5 (24.3 ÷ 34)	0.061 †
EF (%)	62.23 ± 10.305 64.7 (58.63 ÷ 69.33)	63.48 ± 9.255 65.7 (59.1 ÷ 70)	60.29 ± 12.304 64.2 (54.25 ÷ 68.5)	63.33 ± 6.828 64 (58.9 ÷ 69.2)	0.636
ESV (mL)	35.01 ± 15.006 33 (24.1 ÷ 38.2)	35.54 ± 13.831 33.85 (26.65 ÷ 40.48)	36.91 ± 17.772 33 (27.4 ÷ 45.9)	26.76 ± 7.245 24.1 (22.3 ÷ 36.4)	0.279
EDV (mL)	103.73 ± 40.682 98.8 (79.5 ÷ 111.2)	104.81 ± 34.466 98.8 (79.5 ÷ 110.83)	110.42 ± 50.726 101.85 (75.83 ÷ 121.38)	78.11 ± 17.778 80.4 (69.2 ÷ 87.7)	0.093 †
IVSTd (mm)	11.56 ± 1.09 11.85 (11.1 ÷ 12.2)	11.5 ± 1.199 11.8 (11.1 ÷ 12.2)	11.8 ± 0.911 12.1 (11.13 ÷ 12.28)	10.99 ± 1.032 11.3 (10 ÷ 11.8)	0.168
IVSTs (mm)	16.77 ± 2.489 17 (15.15 ÷ 18.3)	16.95 ± 2.5 17 (14.7 ÷ 18.4)	17.23 ± 2.201 17.05 (15.95 ÷ 18.2)	14.46 ± 2.434 15.4 (12.3 ÷ 16.2)	0.045 *
	Statistically significant differences ‡ between: GOLD stages 3 vs. 4 (*p* = 0.046)				
LVIDd (mm)	45.61 ± 4.47 45.9 (43.93 ÷ 48.1)	46.22 ± 4.098 46.5 (42.8 ÷ 48.1)	45.91 ± 4.237 45.75 (44.4 ÷ 49.08)	41.9 ± 5.657 44.7 (34.6 ÷ 45.3)	0.093 †
LVIDs (mm)	29.48 ± 3.552 29.6 (27.18 ÷ 32.1)	29.77 ± 3.395 29.9 (27.2 ÷ 32.3)	30.07 ± 3.45 30.2 (28.25 ÷ 32.1)	26.14 ± 3.191 26.9 (22.9 ÷ 28.3)	0.034 *
	Statistically significant differences ‡ between: GOLD stages 3 vs. 4 (*p* = 0.032)				
RA (mm)	39.6 ± 7.532 38 (34 ÷ 44)	39.89 ± 6.206 39 (34.5 ÷ 44)	39.95 ± 9.612 38 (34.55 ÷ 44)	36.7 ± 6.324 35.0	0.576
RV (mm)	33.52 ± 9.235 31.55 (29.05 ÷ 35.85)	32.69 ± 4.874 34 (27.7 ÷ 37.35)	34.64 ± 12.9 31.3 (30.63 ÷ 34)	32.2 ± 5.981 31.5	0.911
LVOT diam (mm)	20.06 ± 2.911 19.3 (18.5 ÷ 21.6)	19.8 ± 2.342 18.9 (18.2 ÷ 21.7)	21.26 ± 3.487 20.1 (19.3 ÷ 21.8)	17.2 ± 1.744 16.4	0.040 *
	Statistically significant differences ‡ between: GOLD stages 3 vs. 4 (*p* = 0.043)				

† Kruskal–Wallis test with Bonferroni-adjusted pairwise post hoc tests; ‡ ANOVA test with Tukey HSD post hoc tests; * statistically significant (*p* < 0.05); IQR—interquartile range; LA Vol—left atrium volume; LA H—left atrium height; LA W—left atrium width; LA D—left atrium diameter; EF—ejection fraction; ESV—end-systolic volume; EDV—end-diastolic volume; IVSTd—interventricular septal thickness in diastole; IVSTs—interventricular septal thickness in systole; LVIDd—left ventricular internal diameter in diastole; LVIDs—left ventricular internal diameter in systole; RA—right atrium; RV—right ventricle; LVOT diam—left ventricular outflow tract diameter.

**Table 3:** Echocardiographic characteristics of the study population by sex.

Characteristics	Males Mean ± SD/Median (IQR)	Females Mean ± SD/Median (IQR)	*p* Value
LA Vol (mL)	37.1 ± 14.033 38 (26.95 ÷ 45.6)	34.05 ± 15.363 30.8 (25.05 ÷ 43.1)	0.380
LA H (mm)	47.22 ± 7.089 47.6 (42.6 ÷ 52.4)	45.74 ± 8.469 43 (39.45 ÷ 52.4)	0.470
LA W (mm)	38.81 ± 5.618 38.4 (34.55 ÷ 43.15)	37.78 ± 7.228 38.2 (31.1 ÷ 41.95)	0.537
LA D (mm)	36.22 ± 8.098 34.6 (30.75 ÷ 39.4)	33.47 ± 8.325 33.4 (27.95 ÷ 36.7)	0.225
EF (%)	59.96 ± 11.527 61.7 (54.5 ÷ 68.9)	66.66 ± 5.201 67.8 (64.2 ÷ 69.95)	0.021 *
ESV (mL)	37.14 ± 16.72 34.7 (28.13 ÷ 41.9)	31.15 ± 10.561 29.8 (22 ÷ 36.4)	0.122
EDV (mL)	107.67 ± 45.808 98.8 (81.75 ÷ 115.08)	96.61 ± 28.897 93.4 (73 ÷ 110.95)	0.397
IVSTd (mm)	11.79 ± 0.862 11.9 (11.2 ÷ 12.3)	11.11 ± 1.351 11.7 (10 ÷ 12.1)	0.119
IVSTs (mm)	17.16 ± 2.448 17 (15.5 ÷ 18.3)	16.02 ± 2.453 17 (14.15 ÷ 18.1)	0.341
LVIDd (mm)	45.9 ± 4.254 46.1 (44.7 ÷ 48.15)	45.06 ± 4.926 45.6 (41.6 ÷ 47.8)	0.430
LVIDs (mm)	29.79 ± 3.445 29.9 (27.4 ÷ 32.3)	28.86 ± 3.761 29.3 (25.7 ÷ 31.1)	0.422
LVPWTd (mm)	12.2 ± 1.752 11.7 (11.2 ÷ 12.8)	11.19 ± 1.364 11.1 (10.3 ÷ 12.3)	0.039 *
LVPWTs (mm)	21.8 ± 39.979 15.8 (14.2 ÷ 17.4)	13.85 ± 2.174 14.1 (12.35 ÷ 15.2)	0.006 **
LV MASS d (g)	251.37 ± 63.887 251 (194 ÷ 295.5)	223.24 ± 78.39 208 (165 ÷ 273.5)	0.134
LV MASS s (g)	247.29 ± 77.574 247 (194 ÷ 302.5)	204.71 ± 83.206 190 (142.5 ÷ 232)	0.018 *
E Vel (cm/s)	50.32 ± 14.629 53.75 (47.13 ÷ 60.4)	56.27 ± 4.953 56.1 (51.68 ÷ 60.1)	0.126
A Vel (cm/s)	55.26 ± 11.27 58.3 (53.18 ÷ 61.2)	60.17 ± 4.325 61.3 (59.7 ÷ 62.4)	0.034 *
RA (mm)	41.73 ± 7.603 40 (36.5 ÷ 44)	36.24 ± 6.325 35 (31.55 ÷ 42.5)	0.044 *
RV (mm)	34.88 ± 10.785 33.25 (30.13 ÷ 37)	30.81 ± 4.014 31 (26.83 ÷ 34)	0.139
LVOT diam (mm)	20.59 ± 2.95 19.7 (18.95 ÷ 21.7)	19.05 ± 2.671 18.5 (17.1 ÷ 20.7)	0.045 *
Aortic annulus diam (mm)	26.98 ± 5.589 28.1 (25.48 ÷ 29.75)	24.98 ± 3.033 24.9 (22.65 ÷ 26.65)	0.007 **

* *p* < 0.05; ** *p* < 0.01. Abbreviations: LA Vol—left atrium volume; LA H—left atrium height; LA W—left atrium width; LA D—left atrium diameter; EF—ejection fraction; ESV—end-systolic volume; EDV—end-diastolic volume; IVSTd—interventricular septal thickness in diastole; IVSTs—interventricular septal thickness in systole; LVIDd—left ventricular internal diameter in diastole; LVIDs—left ventricular internal diameter in systole; LVPWTd—left ventricular posterior wall thickness in diastole; LVPWTs—left ventricular posterior wall thickness in systole; LV MASS d/s—left ventricular mass in diastole/systole; E Vel—early diastolic mitral inflow velocity; A Vel—late diastolic mitral inflow velocity; R - right atrium; RV—right ventricle; LVOT diam—left ventricular outflow tract diameter; Ao annulus diam—aortic annulus diameter.

**Table 4:** Inflammatory biomarkers according to the presence of sinus rhythm abnormalities.

Biomarker	Absent (n = 80)			Present (n = 20)			*p* Value
	Mean ± SD	Median	IQR	Mean ± SD	Median	IQR (Q1–Q3)	
**IL-6**	60.5 ± 39.204	42.84	33.67–90.78	50.64 ± 57.621	39.65	28.28–48.63	0.178
**IL-8**	159.44 ± 118.456	125.34	76.98–229.41	145.16 ± 149.701	87.38	46.88–197.96	0.234
**IL-1β**	57.07 ± 145.741	10.22	5.71–18.56	29.71 ± 67.653	8.63	5.45–15.87	0.669
**TNF-α**	16.45 ± 0.562	16.31	16.09–16.63	16.94 ± 2.307	16.51	16.13–16.87	0.284
**SII**	467.7 ± 537.719	378.38	237.06–532.91	644.41 ± 390.097	542.08	393.15–725.59	0.001 **
**SIRI**	1.36 ± 1.464	0.81	0.54–1.30	1.31 ± 1.193	1.01	0.72–1.45	0.173
**MLR**	0.28 ± 0.181	0.24	0.14–0.41	0.28 ± 0.203	0.24	0.18–0.31	0.750
**NLR**	2.23 ± 1.914	1.91	1.31–2.07	2.48 ± 1.171	2.30	1.72–3.00	0.033 *
**LHR**	0.07 ± 0.086	0.04	0.02–0.08	0.04 ± 0.013	0.03	0.03–0.04	0.199
**PLR**	106.71 ± 74.319	102.30	66.02–138.34	143.17 ± 70.216	127.89	100.33–161.04	0.114
**CRP**	2.92 ± 4.179	0.88	0.35–5.47	0.93 ± 1.265	0.62	0.25–1.10	0.163
**ESR**	21.95 ± 15.903	15.50	9.75–28.25	16.45 ± 11.587	12.00	9.00–20.75	0.150
**Fibrinogen**	439.5 ± 100.981	450.50	369.50–550.00	429.56 ± 147.051	411.00	361.50–506.50	0.688

* *p* < 0.05 (statistically significant); ** *p* < 0.01 (statistically highly significant); Abbreviations: IQR—interquartile range; IL—interleukin; TNF-α—tumor necrosis factor-alpha; SII—systemic immune-inflammation index; SIRI—systemic inflammation response index; MLR—monocyte-to-lymphocyte ratio; NLR—neutrophil-to-lymphocyte ratio; LHR—lymphocyte-to-high-density lipoprotein ratio; PLR—platelet-to-lymphocyte ratio; CRP—C-reactive protein; ESR—erythrocyte sedimentation rate.

**Table 5:** ROC analysis—sinus rhythm abnormalities.

Biomarker	AUC	*p*	95% CI Lower	95% CI Upper	Cut-Off Value	Sensitivity	Specificity
**SII**	0.736	0.000	0.613	0.858	443.52	0.713	0.700
**NLR**	0.654	0.040	0.507	0.802	2.09	0.600	0.800

SII—systemic immune-inflammation index, NLR—neutrophil-to-lymphocyte ratio; AUC—area under the curve; CI—confidence interval.

**Table 6:** Inflammatory biomarkers according to the presence of atrial fibrillation/atrial flutter.

Biomarker	Absent (n = 81)			Present (n = 19)			*p* Value
	Mean ± SD	Median	IQR	Mean ± SD	Median	IQR (Q1–Q3)	
**IL-6**	51.35 ± 57.454	40.05	28.48–49.04	58.09 ± 39.331	42.44	32.07–82.33	0.333
**IL-8**	148.3 ± 150.667	93.32	47.13–205.21	146.79 ± 110.866	120.92	76.48–173.72	0.405
**IL-1β**	29.35 ± 67.185	8.18	5.49–15.74	60.44 ± 150.208	10.79	5.47–19.73	0.554
**TNF-α**	16.94 ± 2.289	16.54	16.14–16.87	16.42 ± 0.571	16.28	16.07–16.51	0.173
**SII**	642.84 ± 387.907	541.88	393.23–722.22	465.07 ± 552.323	371.54	231.29–538.05	<0.001 **
**SIRI**	1.3 ± 1.186	1.01	0.72–1.45	1.38 ± 1.499	0.81	0.52–1.33	0.195
**MLR**	0.28 ± 0.202	0.24	0.18–0.30	0.28 ± 0.186	0.23	0.14–0.46	0.651
**NLR**	2.48 ± 1.165	2.30	1.73–3.00	2.23 ± 1.966	1.91	1.27–2.04	0.032 *
**LHR**	0.04 ± 0.013	0.03	0.03–0.04	0.07 ± 0.087	0.04	0.03–0.08	0.113
**PLR**	143.45 ± 69.819	128.50	100.66–163.12	103.62 ± 75.028	99.36	64.95–133.75	0.061 +
**CRP**	0.92 ± 1.26	0.62	0.25–1.10	3.07 ± 4.241	0.89	0.36–5.69	0.083 +
**ESR**	16.36 ± 11.544	12	9.00–20.50	22.63 ± 16.035	16.00	12.00–29.00	0.088 +
**Fibrinogen**	429.56 ± 147.051	411	361.50–506.50	439.5 ± 100.981	450.50	369.50–550.00	0.688

* *p* < 0.05 (statistically significant); ** *p* < 0.01 (statistically highly significant); + *p* < 0.10 (trend toward significance). Abbreviations: IQR—interquartile range;IL—interleukin; TNF-α—tumor necrosis factor-alpha; SII—systemic immune-inflammation index; SIRI—systemic inflammation response index; MLR—monocyte-to-lymphocyte ratio; NLR—neutrophil-to-lymphocyte ratio; LHR—lymphocyte-to-high-density lipoprotein ratio; PLR—platelet-to-lymphocyte ratio; CRP—C-reactive protein; ESR—erythrocyte sedimentation rate.

**Table 7:** ROC analysis—atrial fibrillation/atrial flutter.

Biomarker	AUC	*p*	95% CI Lower	95% CI Upper	Cut-Off Value	Sensitivity	Specificity
**SII (low values)**	0.745	0.000	0.619	0.870	443.52	0.737	0.716
**NLR (low values)**	0.659	0.042	0.505	0.812	2.06	0.789	0.593

SII—systemic immune-inflammation index, NLR—neutrophil-to-lymphocyte ratio; AUC—area under the curve; CI—confidence interval.

**Table 8:** Inflammatory biomarkers according to the presence of VPCs.

Biomarker	Absent (n = 21)			Present (n = 79)			*p* Value
	Mean ± SD	Median	IQR	Mean ± SD	Median	IQR (Q1–Q3)	
**IL-6**	29.85 ± 13.965	25.69	23.29–40.85	56.24 ± 57.511	40.85	30.70–64.78	0.014 *
**IL-8**	184.77 ± 120.566	148.37	106.15–266.11	142.16 ± 146.647	81.44	47.13–166.91	0.086 +
**IL-1β**	42.22 ± 83.524	7.31	4.53–20.26	34.06 ± 89.619	9.64	5.53–15.74	0.635
**TNF-α**	17.09 ± 1.642	16.60	16.13–17.79	16.81 ± 2.154	16.45	16.13–16.77	0.553
**SII**	425.36 ± 355.359	358.25	174.84–619.34	646.69 ± 432.004	538.72	402.74–704.18	0.007 **
**SIRI**	0.78 ± 0.368	0.63	0.48–1.15	1.42 ± 1.33	1.01	0.72–1.56	0.011 *
**MLR**	0.21 ± 0.086	0.19	0.16–0.24	0.29 ± 0.212	0.26	0.19–0.32	0.026 *
**NLR**	1.86 ± 0.984	1.92	0.95–2.55	2.55 ± 1.383	2.19	1.71–3.00	0.037 *
**LHR**	0.05 ± 0.04	0.04	0.03–0.05	0.04 ± 0.042	0.03	0.03–0.05	0.253
**PLR**	120 ± 98.964	102.07	81.71–148.56	139.13 ± 65.689	127.27	99.53–161.76	0.049 *
**CRP**	1.39 ± 1.85	0.72	0.21–1.43	1.31 ± 2.387	0.63	0.31–1.10	0.938
**ESR**	16.88 ± 11.056	15.00	9.00–20.50	17.69 ± 13.034	14.00	9.00–23.00	1.000
**Fibrinogen**	399.33 ± 103.016	379.00	—	435.5 ± 137.564	429.00	367.50–514.00	0.498

* *p* < 0.05 (statistically significant); ** *p* < 0.01 (statistically highly significant); + *p* < 0.10 (trend toward significance). Abbreviations: IQR—interquartile range; IL—interleukin; TNF-α—tumor necrosis factor-alpha; SII—systemic immune-inflammation index; SIRI—systemic inflammation response index; MLR—monocyte-to-lymphocyte ratio; NLR—neutrophil-to-lymphocyte ratio; LHR—lymphocyte-to-high-density lipoprotein ratio; PLR—platelet-to-lymphocyte ratio; CRP—C-reactive protein; ESR—erythrocyte sedimentation rate.

**Table 9:** ROC analysis—VPCs.

Biomarker	AUC	*p*	95% CI Lower	95% CI Upper	Cut-Off Value	Sensitivity	Specificity
**IL-6**	0.732	0.005	0.569	0.895	26.09	0.855	0.545
**SII**	0.682	0.064	0.489	0.876	364.94	0.797	0.636
**SIRI**	0.702	0.012	0.545	0.859	0.63	0.826	0.545
**MLR**	0.673	0.022	0.525	0.822	0.25	0.493	0.919
**NLR**	0.635	0.129	0.460	0.810	1.48	0.899	0.364
**PLR**	0.648	0.142	0.450	0.846	87.11	0.884	0.455

IL—interleukin; SII—systemic immune-inflammation index; SIRI—systemic inflammation response index; MLR—monocyte-to-lymphocyte ratio; NLR—neutrophil-to-lymphocyte ratio; PLR—platelet-to-lymphocyte ratio; AUC—area under the curve; CI—confidence interval.

**Table 10:** Inflammatory biomarkers according to the presence of isolated supraventricular ectopic beats.

Biomarker	Absent (n = 18)			Present (n = 82)			*p* Value
	Mean ± SD	Median	IQR	Mean ± SD	Median	IQR (Q1–Q3)	
**IL-6**	29.84 ± 13.315	27.69	23.29–40.45	56.64 ± 57.846	40.85	31.80–65.57	0.009 **
**IL-8**	173.75 ± 121.121	134.65	86.88–264.20	143.47 ± 147.326	86.39	46.88–170.31	0.155
**IL-1β**	38.89 ± 80.468	6.76	4.40–18.07	34.52 ± 90.2	9.87	5.62–15.87	0.356
**TNF-α**	17.03 ± 1.576	16.53	16.14–17.59	16.81 ± 2.169	16.43	16.13–16.78	0.567
**SII**	375 ± 209.166	358.29	188.70–569.85	660.44 ± 445.4	539.07	400.32–719.60	0.002 **
**SIRI**	0.77 ± 0.349	0.70	0.49–1.06	1.44 ± 1.336	1.04	0.72–1.56	0.005 **
**MLR**	0.21 ± 0.08	0.19	0.16–0.24	0.3 ± 0.213	0.26	0.19–0.32	0.013 *
**NLR**	1.75 ± 0.787	1.81	0.96–2.36	2.58 ± 1.398	2.19	1.74–3.10	0.011 *
**LHR**	0.05 ± 0.039	0.04	0.03–0.05	0.04 ± 0.042	0.03	0.02–0.04	0.116
**PLR**	104.21 ± 48.459	104.21	81.76–147.06	142.83 ± 74.847	127.89	99.48–162.44	0.052 +
**CRP**	1.29 ± 1.766	0.58	0.21–1.43	1.33 ± 2.406	0.64	0.31–1.10	0.928
**ESR**	18.5 ± 14.267	16.00	9.00–26.00	17.34 ± 12.38	13.50	9.00–22.25	0.872
**Fibrinogen**	304.4 ± 187.523	323.00	154.50–445.00	453.73 ± 113.748	438.00	378.00–525.75	0.056 +

* *p* < 0.05 (statistically significant); ** *p* < 0.01 (statistically highly significant); + *p* < 0.10 (trend toward significance). Abbreviations: IQR—interquartile range; IL—interleukin; TNF-α—tumor necrosis factor-alpha; SII—systemic immune-inflammation index; SIRI—systemic inflammation response index; MLR—monocyte-to-lymphocyte ratio; NLR—neutrophil-to-lymphocyte ratio; LHR—lymphocyte-to-high-density lipoprotein ratio; PLR—platelet-to-lymphocyte ratio; CRP—C-reactive protein; ESR—erythrocyte sedimentation rate.

**Table 11:** ROC analysis—isolated supraventricular ectopic beats.

Biomarker	AUC	*p*	95% CI Lower		Cut-Off Value	Sensitivity	Specificity
			Lower	Upper			
IL-6	0.738	0.002	0.587	0.889	33.27	0.735	0.667
SII	0.659	0.090	0.475	0.843	364.94	0.794	0.583
SIRI	0.692	0.011	0.544	0.841	1.20	0.338	1.000
MLR	0.643	0.059	0.495	0.792	0.25	0.485	0.833
NLR	0.615	0.177	0.448	0.783	2.86	0.265	1.000

IL—interleukin; SII—systemic immune-inflammation index; SIRI—systemic inflammation response index; MLR—monocyte-to-lymphocyte ratio; NLR—neutrophil-to-lymphocyte ratio; AUC—area under the curve; CI—confidence interval.

**Table 12:** Inflammatory biomarkers according to the presence of supraventricular couplets.

Biomarker	Absent (n = 26)			Present (n = 74)			*p* Value
	Mean ± SD	Median	IQR	Mean ± SD	Median	IQR (Q1–Q3)	
**IL-6**	61.03 ± 76.125	40.05	27.28–68.17	46.07 ± 26.979	40.05	28.88–47.63	0.827
**IL-8**	153.02 ± 115.4	118.96	52.61–258.48	144.13 ± 163.11	75.49	46.63–158.60	0.192
**IL-1β**	48.66 ± 116.221	8.18	5.92–20.60	24.69 ± 57.599	9.64	5.35–13.56	0.597
**TNF-α**	16.76 ± 1.028	16.54	16.17–16.86	16.91 ± 2.64	16.36	16.10–16.82	0.308
**SII**	592.63 ± 486.007	515.48	304.18–693.46	621.98 ± 377.486	536.55	395.48–690.34	0.399
**SIRI**	1.2 ± 1.314	0.87	0.53–1.19	1.41 ± 1.189	1.08	0.79–1.56	0.015 *
**MLR**	0.24 ± 0.114	0.20	0.16–0.29	0.31 ± 0.241	0.26	0.19–0.33	0.023 *
**NLR**	2.44 ± 1.717	2.01	1.53–2.74	2.43 ± 0.975	2.19	1.68–3.07	0.274
**LHR**	0.04 ± 0.029	0.03	0.03–0.04	0.04 ± 0.05	0.03	0.03–0.05	0.936
**PLR**	130.65 ± 68.438	117.86	92.90–158.67	139.99 ± 75.336	126.95	97.87–155.26	0.399
**CRP**	1.09 ± 1.465	0.68	0.25–1.10	1.51 ± 2.781	0.61	0.30–1.17	0.903
**ESR**	17.07 ± 11.798	12.00	9.00–22.00	17.93 ± 13.411	14.00	10.00–23.00	0.697
**Fibrinogen**	373.67 ± 149.799	372.00	320.75–478.25	463.04 ± 117.09	446.00	387.00–549.00	0.054 +

* *p* < 0.05 (statistically significant); + *p* < 0.10 (trend toward significance). Abbreviations: IQR—interquartile range;IL—interleukin; TNF-α—tumor necrosis factor-alpha; SII—systemic immune-inflammation index; SIRI—systemic inflammation response index; MLR—monocyte-to-lymphocyte ratio; NLR—neutrophil-to-lymphocyte ratio; LHR—lymphocyte-to-high-density lipoprotein ratio; PLR—platelet-to-lymphocyte ratio; CRP—C-reactive protein; ESR—erythrocyte sedimentation rate.

**Table 13:** Inflammatory biomarkers according to the presence of paroxysmal supraventricular tachycardia (PSVT).

Biomarker	Absent (n = 72)			Present (n = 28)			*p* Value
	Mean ± SD	Median	IQR	Mean ± SD	Median	IQR (Q1–Q3)	
**IL-6**	52.48 ± 55.44	40.05	28.88–49.24	53.66 ± 47.833	40.85	32.07–69.66	0.626
**IL-8**	135.59 ± 133.088	91.34	47.62–173.72	246.04 ± 189.622	160.09	101.96–370.14	0.039 *
**IL-1β**	35.34 ± 91.905	9.08	5.23–15.48	33.87 ± 56.204	12.36	7.40–35.43	0.190
**TNF-α**	16.86 ± 2.186	16.45	16.13–16.84	16.7 ± 1.029	16.41	16.00–17.06	0.703
**SII**	594.6 ± 434.445	512.33	353.02–676.87	715.15 ± 361.954	595.81	453.32–950.17	0.127
**SIRI**	1.26 ± 1.235	0.91	0.64–1.37	1.7 ± 1.292	1.39	0.82–1.68	0.066 +
**MLR**	0.27 ± 0.204	0.23	0.18–0.30	0.32 ± 0.146	0.27	0.22–0.45	0.136
**NLR**	2.39 ± 1.367	2.11	1.61–2.88	2.73 ± 1.179	2.60	1.91–3.98	0.254
**LHR**	0.04 ± 0.044	0.03	0.03–0.04	0.05 ± 0.019	0.04	0.03–0.06	0.078 +
**PLR**	133.27 ± 67.937	125.35	96.43–157.44	154.99 ± 99.624	111.52	101.33–205.35	0.890
**CRP**	1.23 ± 2.036	0.65	0.27–1.10	1.99 ± 3.758	0.49	0.26–1.56	0.983
**ESR**	16.68 ± 12.307	12.50	9.00–21.75	23.92 ± 14.029	20.00	12.75–31.75	0.035 *
**Fibrinogen**	425.32 ± 140.279	407.00	358.00–511.00	487.25 ± 50.076	482.00	441.75–538.00	0.169

* *p* < 0.05 (statistically significant); + *p* < 0.10 (trend toward significance). Abbreviations: IQR—interquartile range; IL—interleukin; TNF-α—tumor necrosis factor-alpha; SII—systemic immune-inflammation index; SIRI—systemic inflammation response index; MLR—monocyte-to-lymphocyte ratio; NLR—neutrophil-to-lymphocyte ratio; LHR—lymphocyte-to-high-density lipoprotein ratio; PLR—platelet-to-lymphocyte ratio; CRP—C-reactive protein; ESR—erythrocyte sedimentation rate.

**Table 14:** ROC analysis—PSVT.

Biomarker	AUC	*p*	95% CI Lower	95% CI Upper	Cut-Off Value	Sensitivity	Specificity
**IL-8**	0.712	0.033	0.517	0.907	149.84	0.778	0.746
**ESR**	0.701	0.029	0.520	0.882	18.50	0.667	0.746

ESR—erythrocyte sedimentation rate; AUC—area under the curve; CI—confidence interval.

**Table 15:** Correlation analysis between inflammatory biomarkers and electrocardiographic parameters.

Biomarker	ST Depression		rMSSD		Max QT (ms)		Max QTc (ms)		HRV	
	r	*p*	r	*p*	r	*p*	r	*p*	r	*p*
**IL-8**	−0.550	0.041 *	−0.110	0.374	−0.024	0.845	−0.009	0.942	0.022	0.861
**MLR**	−0.481	0.037 *	−0.181	0.105	0.101	0.368	0.098	0.386	−0.297	0.007 **
**NLR**	0.070	0.777	−0.039	0.733	0.222	0.046 *	0.235	0.035 *	−0.108	0.338
**LHR**	−0.251	0.299	0.001	0.996	−0.246	0.027 *	−0.242	0.029 *	0.169	0.132
**PLR**	0.184	0.450	−0.115	0.307	0.149	0.184	0.176	0.115	−0.228	0.041 *
**Fibrinogen**	−0.008	0.986	−0.433	0.019 *	0.031	0.872	0.049	0.797	−0.286	0.126

r—Pearson/Spearman correlation coefficient. * *p* < 0.05; ** *p* < 0.01. Abbreviations: rMSSD—root mean square of successive differences (heart rate variability parameter); QT—QT interval; QTc—corrected QT interval; HRV—heart rate variability; MLR—monocyte-to-lymphocyte ratio; NLR—neutrophil-to-lymphocyte ratio; LHR—lymphocyte-to-high-density lipoprotein ratio; PLR—platelet-to-lymphocyte ratio.

**Table 16:** Exploratory multivariable logistic regression analyses of SII and NLR in relation to sinus rhythm abnormalities.

Predictor	Model 1—SII aOR (95% CI)	*p*-Value	Model 2—NLR aOR (95% CI)	*p*-Value
**SII, per doubling**	**2.79 (1.36–5.71)**	**0.005**	**–**	**–**
**NLR, per doubling**	**–**	**–**	**2.37 (1.09–5.19)**	**0.030**
Age, per year	0.99 (0.92–1.06)	0.698	0.97 (0.91–1.04)	0.406
Male sex (vs. female)	1.70 (0.50–5.76)	0.396	1.48 (0.46–4.81)	0.511
GOLD stage, per stage	1.80 (0.66–4.89)	0.248	1.98 (0.74–5.28)	0.172
Resting SpO_2_, per 1%	**1.38 (1.06–1.79)**	**0.016**	**1.37 (1.06–1.76)**	**0.017**
**Sensitivity analysis**				
**SII, per doubling**	**2.84 (1.34–6.01)**	**0.006**	**–**	**–**
**NLR, per doubling**	**–**	**–**	**2.49 (1.11–5.60)**	**0.027**

SII and NLR were log_2_-transformed before regression analysis; therefore, aORs are expressed per doubling of the respective biomarker. Primary models were adjusted for age, sex, GOLD stage, and resting SpO_2_. Sensitivity analyses were additionally adjusted for smoking status, chronic ischemic heart disease, and beta-blocker use. Abbreviations: aOR, adjusted odds ratio; CI, confidence interval; SII, systemic immune-inflammation index; NLR, neutrophil-to-lymphocyte ratio; GOLD, Global Initiative for Chronic Obstructive Lung Disease; SpO_2_, peripheral oxygen saturation.

## Data Availability

All data presented in this study are available within the article. The first author has all data used in this study.
